# SIN-3 functions through multi-protein interaction to regulate apoptosis, autophagy, and longevity in *Caenorhabditis elegans*

**DOI:** 10.1038/s41598-022-13864-0

**Published:** 2022-06-22

**Authors:** Chandrika Konwar, Jayant Maini, Surbhi Kohli, Vani Brahmachari, Daman Saluja

**Affiliations:** 1 Dr. B. R. Ambedkar Center for Biomedical Research, University of Delhi, New Delhi, Delhi 110007 India; 2 Delhi School of Public Health, IoE, University of Delhi, New Delhi, Delhi 110007 India; 3 Molecular Biosciences Research Cluster, Department of Biotechnology, Manav Rachna International Institute of Research and Studies, Surajkund Road, Faridabad, Haryana 121004 India

**Keywords:** Regulatory networks, Systems analysis

## Abstract

SIN3/HDAC is a multi-protein complex that acts as a regulatory unit and functions as a co-repressor/co-activator and a general transcription factor. SIN3 acts as a scaffold in the complex, binding directly to HDAC1/2 and other proteins and plays crucial roles in regulating apoptosis, differentiation, cell proliferation, development, and cell cycle. However, its exact mechanism of action remains elusive. Using the *Caenorhabditis elegans* (*C. elegans*) model, we can surpass the challenges posed by the functional redundancy of SIN3 isoforms. In this regard, we have previously demonstrated the role of SIN-3 in uncoupling autophagy and longevity in *C. elegans*. In order to understand the mechanism of action of SIN3 in these processes, we carried out a comparative analysis of the SIN3 protein interactome from model organisms of different phyla. We identified conserved, expanded, and contracted gene classes. The *C. elegans* SIN-3 interactome -revealed the presence of  well-known proteins, such as DAF-16, SIR-2.1, SGK-1, and AKT-1/2, involved in autophagy, apoptosis, and longevity. Overall, our analyses propose  potential mechanisms by which SIN3 participates in multiple biological processes and their conservation across species and identifies candidate genes for further experimental analysis.

## Introduction

Cell death and aging are known to play a crucial role in development, health and disease. Biological processes such as cell death and aging are tightly controlled by gene expression, mediated by complex interactions between chromatin, epigenetic modifiers, and transcription regulatory proteins (TRPs). TRPs such as the SIN3 protein are associated with DNA-binding transcription factors in addition to different co-activator and co-repressor complexes. These protein–protein interactions function as writers, erasers, readers and modifiers of the chromatin design for the functioning of a cell^1,2^. SIN3 protein is a transcriptional regulator that functions as the central scaffold unit of the multi-protein SIN3/HDAC co-repressor complex^3^. This SIN3 core complex and its interaction partners play a significant role in several critical pathways such as autophagy, apoptosis and longevity.

A typical SIN3 protein harbours several paired amphipathic α-helix (PAH) domains, a prominent HDAC interacting domain (HID), and a highly conserved region (HCR)^4^. These evolutionarily conserved domains enable the protein to control transcription^5,6^. Though SIN3 has multiple roles in apoptosis, differentiation, cellular proliferation, development, cell cycle, cancer and aging^7–10^, the exact mechanism of regulation mediated by the protein is unknown.

The functional diversity of SIN3 can be facilitated by the diversity in its interactome. However, the mechanistic studies are impeded by the presence of multiple isoforms of SIN3 in mammals and their functional redundancy. The presence of SIN-3 and the absence of isoforms of SIN3 in *C. elegans* makes it a suitable model for understanding the functional diversity of SIN3.

In *C. elegans*, SIN-3 protein plays a vital role in male sensory organ development^11^, muscle integrity, motility, and longevity. It also controls several physiological parameters such as stress tolerance, protein homeostasis, muscle and mitochondrial functioning, cuticle integrity, accumulation of age-associated pigments, fecundity and fertility^12^. Remarkably, the protein modulates autophagy and lifespan in an unconventional way. Usually, an increase in autophagy leads to lifespan extension. However, reactive oxygen species (ROS) and intracellular oxidative stress in *sin-3* deletion mutants are associated with uncoupling of autophagy and longevity such that increased autophagy leads to a shorter lifespan^13^. In order to understand the factors involved in SIN3 regulated autophagy and longevity, we carried out an analysis of the interactome of SIN3 in different model organisms from different phyla. Further analysis of gene ontology and the expansion/contraction of the gene classes led to the identification of conserved factors important for these functions that can serve as candidate genes for further analysis.

## Methods

### Software and databases

NCBI Protein database, Clustal Omega web server, MAFFT, MEGA-X v10, NCBI Conserved domain database, G-BLOCKS online web server v0.91b, GeneMANIA, DAVID Bioinformatics Resource v6.8, NCBI Conserved domain database, Ensembl genome, Orthofinder (version 2.3.12), RStudio v2021.09.0+351.pro6 (https://www.rstudio.com/)^14^, Microsoft Excel 2013 (https://www.microsoft.com/)^15^.

### Retrieval of sequences and their analysis

The FASTA sequences of SIN3 homologs were downloaded from the NCBI Protein database^16^ and submitted to the Clustal Omega web server (http://www.ebi.ac.uk/Tools/msa/clustalo/) for proper sequence alignment and domain comparison^17–19^. The percent identity matrix was obtained for all the SIN3 homologs. Similarly, the percent identity matrix for SIN3 protein domains, such as PAH, SIN3A_C, and HDAC interaction domain, was also prepared for domain-specific comparison.

### Phylogenetic analysis

The FASTA sequences of SIN3 homologs were downloaded and aligned in the MAFFT (L-INS-I method) online web server v7^20^. L-INS-i is one of the most accurate multiple sequence alignments, particularly suitable to align 10–100 protein sequences. The amino acid alignment was curated in the G-BLOCKS online web server v0.91b^21^ with default parameters (except minimum block length set as 5 and allowed gap positions set as half). Phylogenetic tree was constructed through Maximum likelihood (ML) using MEGA-X v10^22^. Statistical significance was increased by bootstrapping with 1000 replicates^23^. The phylogenetic tree for SIN3 protein domains, PAH, SIN3A_C and HID, was obtained in the same manner.

### Domain search

The FASTA sequences of SIN3 homologs were submitted to the NCBI Conserved domain database^24^ and analyzed for the different functional domains present in the SIN3 proteins.

### Protein–protein interaction network

GeneMANIA (http://Genemania.org) was used to examine the various physical interactions of SIN3 homologs with a limitation on the maximum resultant genes, set to 100^25^. After selecting the specific organism under study, the official gene symbol of the protein of interest was submitted to the database. All default network attributes were de-selected, and only physical interactions were selected for generating the final SIN3 protein interactome.

### Gene ontology analysis

DAVID (the information base for Annotation, Visualization, and Integrated Discovery) Bioinformatics Resource v6.8 was used for analyzing the physical protein interactors of *C. elegans* SIN-3^26–28^. First, the gene list was uploaded to the database, and the saved list was then used as the input for the Functional Annotation tool. Finally, the protein list was separately used to extract and summarize the KEGG pathways^29^, InterPro domains^30^, and Gene ontology^31^ of the SIN-3 protein interactors using the different parameters.

### Orthology

For evaluating the orthology of proteins of interest, the predicted proteomes of *C. elegans* (WBcel235), *S. cerevisiae* (R64-1-1), *D. melanogaster* (BDGP6.32), *D. rerio* (GRCz11), *H. sapiens* (GRCh38.p13), and *M. musculus* (GRCm39) from the Ensembl genome (ensembl.org) were used. Orthofinder (version 2.3.12) was run with default parameters to find orthogroups among the whole deduced proteomes of all six organisms listed above^32^. If there are multiple predicted proteins/transcripts for a gene, the primary transcript of the predicted protein was chosen for analysis.

## Results and discussion

SIN3 is well-conserved across phyla due to the significant similarities in the functional domains of the protein. These domains and other protein motifs enable SIN3 to participate in multiple protein–protein interactions, hence regulating multiple pathways. The analysis we have carried out identifies candidate protein–protein interactions of SIN3 involved in the eukaryotic regulation of crucial biological processes like apoptosis, autophagy and longevity.

### *ceSIN3* shares sequence identity with homologs

**Figure 1:**
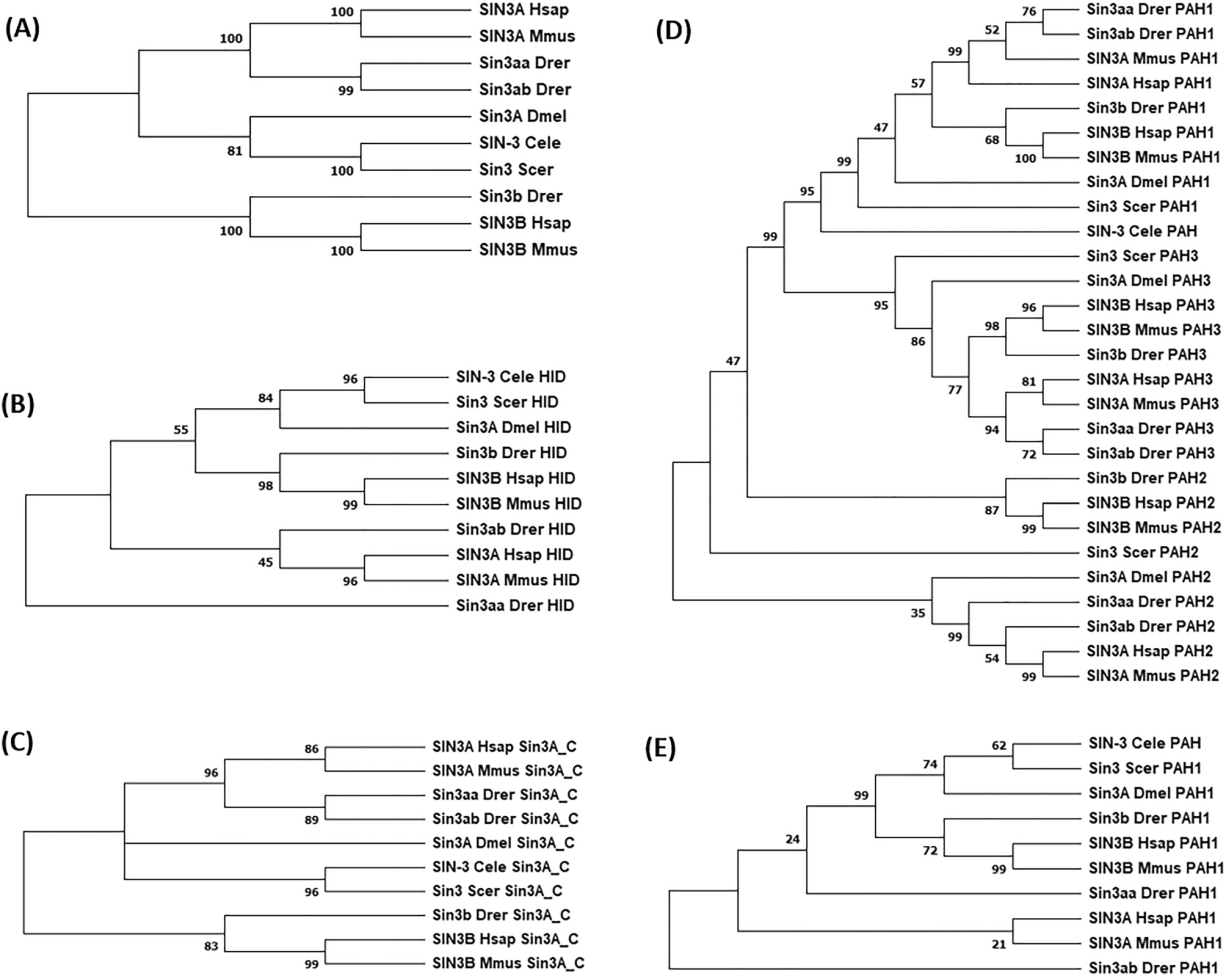
SIN3 phylogeny based on alignments of the amino-acid sequences of *Caenorhabditis elegans* SIN-3 protein and its domains. Phylogenetic tree based on (**A**) SIN3 protein, (**B**) SIN3 HID domain, (**C**) SIN3 SIN3A_C domain, (**D**) SIN3 PAH domain, and (**E**) SIN3 PAH1 domain. The PAH1 domain was analysed separately due to its high sequence identity with PAH domain of *C. elegans* SIN-3 protein. The Phylogenetic tree was constructed using the Maximum Likelihood method of phylogenetic tree construction, with 1000 bootstrap replicates, using MEGA-X. The number mentioned at each node is the bootstrap percentage for a particular branch (Cele, *Caenorhabditis elegans;* Hsap, *Homo sapiens*; Mmus, *Mus musculus*; Drer, *Danio rerio*; Dmel, *Drosophila melanogaster*; Scer, *Saccharomyces cerevisiae*).

One of the main approaches to understanding SIN3 function is the study of protein conservation determined via sequence homology between *C. elegans* SIN-3 protein (*ceSIN3*) and SIN3 isoforms present in other model organisms. Previous studies have reported that *ceSIN3* has homology with human and mouse SIN3^11,13^. Therefore, we carried out phylogenomic analyses of the SIN-3 protein of *C. elegans* and five other model organisms, namely, *S. cerevisiae, D. melanogaster, D. rerio, H. sapiens,* and *M. musculus,* to deduce the level of protein homology in the context of the functional domains. *ceSIN3* is an orthologous member of the SIN3A phylogenetic family (Fig. 1A). Traditionally, *C. elegans* shares a close evolutionary relationship with *Drosophila*, followed by the yeast^33^. This is reflected in the phylogenetic tree of the SIN3 protein, which shows that SIN3 homologs of *C. elegans*, *Drosophila*, and yeast form a separate cluster within the SIN3A family, indicating high relatedness in the model organisms with lower complexity.

To determine the quantitative similarity, a percent identity (PID) analysis was carried out using ClustalW sequence alignment. *ceSIN3* protein has a significant identity, ~ 21%, with all SIN3 isoforms present in different organisms that we analysed (Table 1, Fig. S1). It is generally believed that two sequences are homologous only if there is more than 30% identity at the protein sequence level^34^. However, the sequence length determines the possibility of correct alignment, and if proteins with large sequence length exhibit ≥ 20% identity, they are considered significant even in the twilight zone of evolutionary relatedness (20–30%)^35^. Therefore, the current study demonstrates that *ceSIN3* protein shares homology with human and mice SIN3. It also shows significant similarity with the SIN3 isoforms of *S. cerevisiae*, *D. melanogaster*, and *D. rerio*.

**Table 1:** Percentage sequence identity of *C. elegans* SIN-3 protein with the SIN3 isoforms in other model organisms (Hsap, *Homo sapiens*; Mmus, *Mus musculus*; Drer, *Danio rerio*; Dmel, *Drosophila melanogaster*; Scer, *Saccharomyces cerevisiae*).

	Scer Sin3	Dmel Sin3A	Hsap SIN3A	Hsap SIN3B	Mmus SIN3A	Mmus SIN3B	Drer Sin3aa	Drer Sin3ab	Drer Sin3b
*C. elegans* SIN-3	20.33	21.62	22.2	22.14	22	22.69	21.53	21.14	22.38

However, the multiple sequence alignment indicates that the sequence identity of *ceSIN3* with the rest of the proteins is restricted to specific regions. While the PAH and HID domains contain the well-aligned regions of the protein, it was also seen that the *C. elegans* protein has large stretches of unaligned regions that seem to be only partially conserved in SIN3 proteins of yeast and *Drosophila* (Fig. S2). The close phylogenetic relationship among SIN3 homologs of these organisms could arise from the conservation of these specific regions. Interestingly, the nematode protein shares a closer relationship with SIN3A isoforms than SIN3B even though it shares the same sequence identity with both the isoforms (Fig. 1A). This might be because SIN3A and SIN3B are paralogs that evolved out of gene duplication. While SIN3A retained its similarity to the ancestral lineage, SIN3B underwent substantial diversification since its origin.

### SIN3 homology for PAH and HID

SIN3 protein has multiple protein partners that interact through several structural and functional domains. In order to understand the nature of conservation in *ceSIN3* protein, these unique domains were also analyzed for their sequence homology.

Earlier studies on SIN3 by Chaubal and Pile^36^ indicated the presence of PAH, HID and SIN3A_C domains in the isoforms of *C. elegans*, *Drosophila*, zebrafish and mouse. Our analysis supports this finding and further discloses that *CeSIN3* protein is the only SIN3 homolog to harbor a single PAH domain. This domain has homology with all the three PAH domains of SIN3 isoforms. The *CeSIN3* protein also contains one HDAC interaction domain and one SIN3A_C domain (Table 2).

**Table 2:** Conservation of SIN-3 protein domains of *C. elegans* with those of other organisms expressed in percent similarity. *Scer* (*Saccharomyces cerevisiae*), *Dmel* (*Drosophila melanogaster*), *Mmus* (*Mus musculus*), *Hsap* (*Homo sapiens*), *Drer* (*Danio rerio*). *Caenorhabditis elegans* SIN-3 protein consists of a single PAH domain.

Domain	Scer	Dmel	Mmus		Hsap		Drer		
	Sin3	Sin3A	SIN3A	SIN3B	SIN3A	SIN3B	Sin3aa	Sin3ab	Sin3b
PAH1	55.56	48.89	53.33	51.11	53.33	51.11	53.33	53.33	54.55
PAH2	31.11	33.33	31.11	31.11	31.11	31.11	29.55	33.33	31.11
PAH3	27.91	26.67	28.89	28.89	28.89	28.89	28.89	28.89	28.89
HID	38.14	39.18	39.58	39.58	39.58	39.39	40.62	40.62	40.62
SIN3A_C	19.08	18.5	20.77	19.67	20.77	18.58	18.03	18.03	19.57

We found unique motifs, a large stretch of Glutamine rich repeats, and two interspersed regions of Aspartate rich repeats in the *ceSIN3* protein (Fig. 3). These amino acid repeats seem to coincide with the unaligned regions of *ceSIN3* (Table S2). These repeats are absent in other SIN3 homologs except yeast Sin3 protein, which contains a big stretch of Glutamine rich repeats. While excessive repeat expansion is pathogenic, Glutamine-rich repeats and Aspartate or Glutamate repeat with regular lengths help in gene regulation and protein–protein interaction^37,38^. Hence, *ceSIN3* protein could participate in specific protein interactions involved in biological processes unique to the organism.

Proteins and transcription factors binding to the PAH domain in yeast or mammalian systems might also bind to *C. elegans* SIN-3 PAH (*cePAH*). The current study reveals that the PAH domain of *ceSIN3* has ≥ 28% identity with all the three PAH domains of SIN3 isoforms. The *cePAH* domain is remarkably similar to the PAH1 (≥ 50%) domain of the other SIN3 homologs, especially the yeast Sin3 PAH1 (Table 2, Fig. S2). Surprisingly, despite the sequence homology of the *cePAH* domain, the protein domain is phylogenetically distant from all the three PAH domains, forming an out-group in the cluster containing PAH1 domains (Fig. 1D). But when the domain is studied exclusively in relation to PAH1 domains, it shows a close phylogenetic relationship with yeast Sin3 PAH1 domain (Fig. 1E).

Similarly, *C. elegans* HDAC interaction domain shows 38.14–40.62% sequence identity with all SIN3 homologs HDAC domains, principally the SIN3 isoforms in zebrafish (Table 2). However, it shares the closest phylogenetic relationship with the yeast Sin3 protein (Fig. 1B). In addition, *C. elegans* HDAC interaction domain has almost perfect sequence alignment with its homologs (Fig. S2). Due to this alignment, a good percentage of identity exists between the HDAC interaction domain of *ceSIN3* protein and homologs. Unlike HID and PAH domains, the percent identity matrix and poor sequence alignment of the evolutionarily well-conserved SIN3A_C domain hint at a low sequence identity between the nematode protein and the other SIN3 homologs (Table 2, Fig. S2). This also accounts for the phylogenetic relationship between SIN3A_C domains of the worm and yeast (Fig. 1C). Overall, the conservation in *CeSIN3* protein seems to be localized in the PAH and HID domains.

### Orthologs in *C. elegans* genome

**Figure 2:**
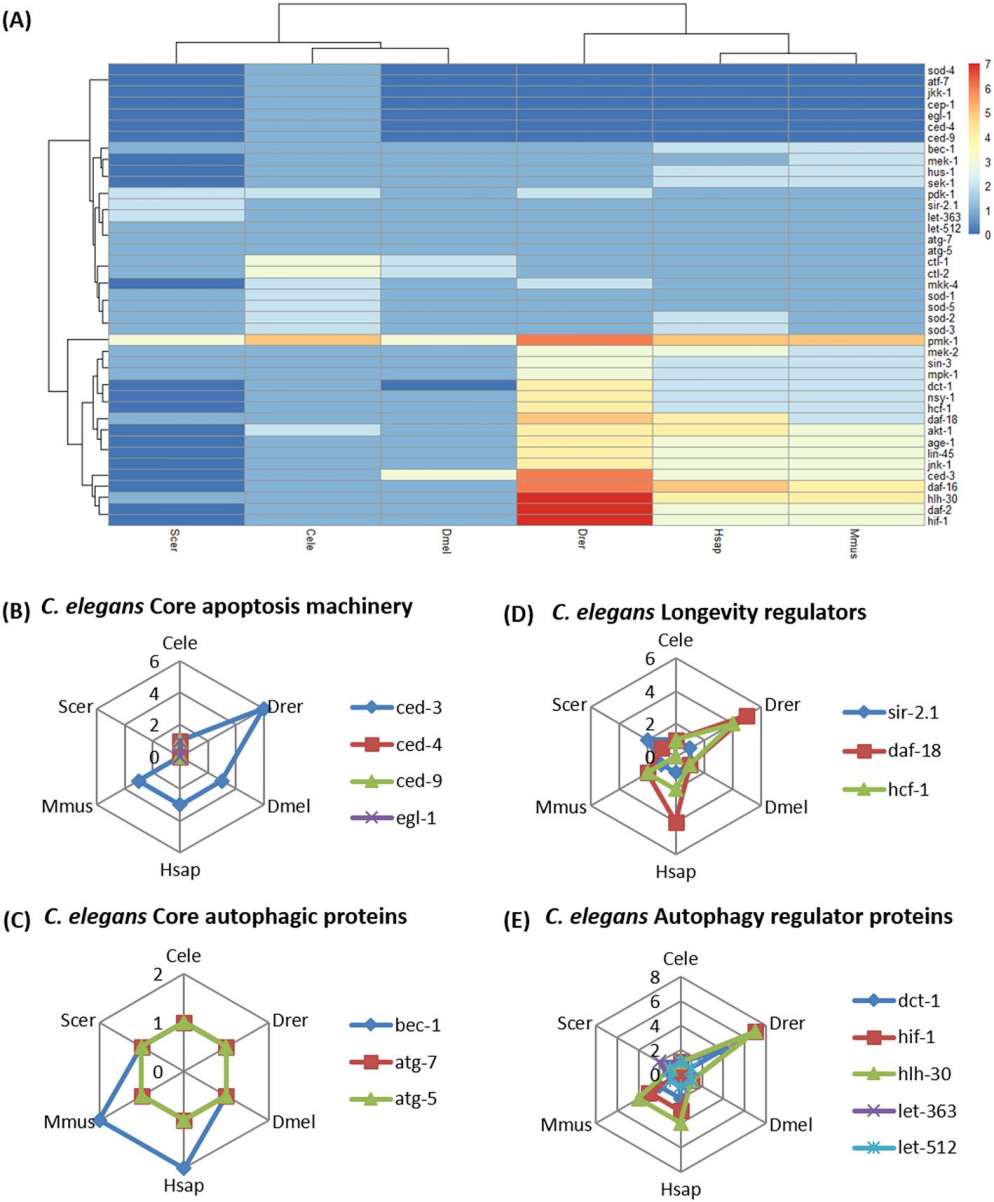
Abundance of proteins involved in apoptosis, autophagy and longevity in *C. elegans.* (**A**) Heatmap depicting the orthology of 41 proteins derived from 18,002 comparative analyses of gene classes using OrthoFinder, in the proteome of *Caenorhabditis elegans (*Cele*), Danio rerio (Drer), Drosophila melanogaster (Dmel), Homo sapiens (Hsap), Mus musculus (Mmus),* and *Saccharomyces cerevisiae (Scer).* Color scale key is depicted at the side. The spider plots further indicate the number of proteins in the different species; *C. elegans* specific pathways like core apoptosis machinery (**B**), longevity regulators (**C**), core autophagic proteins (**D**) and autophagic regulator proteins) (**E**) are indicated at the top of each spider plot.

The gain or loss of gene function or its regulation contributes to the adaptation and survival of organisms. As the study focuses on understanding SIN3 mediated regulation of apoptosis, autophagy and longevity, the orthology of the major nematode proteins associated with these crucial biological processes was examined. The data derived from 18,002 comparative analyses of proteins using OrthoFinder, that are over-represented or under-represented in the proteome of *C. elegans*, Yeast, *Drosophila*, Zebrafish, Human, and Mouse was used to construct orthogroups (Fig. 2). Of the 41 proteins investigated, we identified 34 different orthogroups having conservation, expansion, and contraction and 7 single copy orthologue sequences (Fig. 2, Fig. S3).

Protein orthogroups involved in signaling pathways are well conserved in higher eukaryotes. While the number of proteins attributed to the different orthogroups is similar for the invertebrates, *C. elegans* and *Drosophila*, proteins involved in important pathways such as the ERK-MAPK pathway (LIN-45, MEK-2, MPK-1), p38-MAPK pathway (NSY-1, PMK-1), and insulin signaling pathway (DAF-2, DAF-16, AGE-1, AKT-1) are conserved across phyla and expanded in proteomes of three or more species. These pathway proteins are crucial for cellular processes preceding apoptosis and longevity, and are expanded in the eukaryotic system. The ERK-MAPK pathway is the primary signaling network involved in regulating cell division, growth, development, apoptosis^39^ and tumor formation, and the p38-MAPK pathway works in response to stress to regulate cellular functions such as differentiation, apoptosis, and senescence^40^. The insulin/IGF signaling (IIS) pathway is involved in stress resistance and longevity along with nutrient regulation and growth^41^. Interestingly, orthogroups of signaling proteins (MKK-4, PDK-1, AKT-1 AND PMK-1) are also expanded in the nematode proteome, revealing the need for increased signaling proteins even in lower eukaryotes.

Orthogroups of nematode proteins that show poor conservation in other model organisms are mainly associated with the main apoptotic pathway. While the main cell death protein CED-3 (caspase 2/3/6 homolog) protein is expanded in higher eukaryotes, rest of the apoptosis proteins (CED-4, CED-9, EGL-1, CEP-1) are contracted in the species included in the study. This is likely because of the functional redundancy of the proteins associated with essential functions. For example, CEP-1, a functional p53 homolog, has very low sequence identity with p53 isoforms of different model organisms^42^.

Most of the orthogroups do not have any yeast protein as the unicellular organism lacks homologs for most eukaryotic proteins involved in apoptosis, autophagy, and longevity. Yeast does not have distinct cell death pathways similar to mammalian ones^43^; even the MAPK cascade components are only conserved in function^44^ but not in terms of sequence identity. Certain zebrafish proteins are over-represented due to genome duplication, which precedes the teleost evolution. As a result, many mammalian genes have more than one ortholog in the zebrafish genome^45,46^.

### Protein–protein interactions in the SIN3 network

Protein–protein interactions are crucial for the basic functionality of cells (40). The detailed study of the protein interactome holds the key to identifying biological pathways and predicting protein functions^47–50^. Therefore, identifying the interaction repertoire of transcriptional regulator SIN-3 will give a better understanding of the regulation of various biological processes in *C. elegans*^51^.

**Figure 3:**
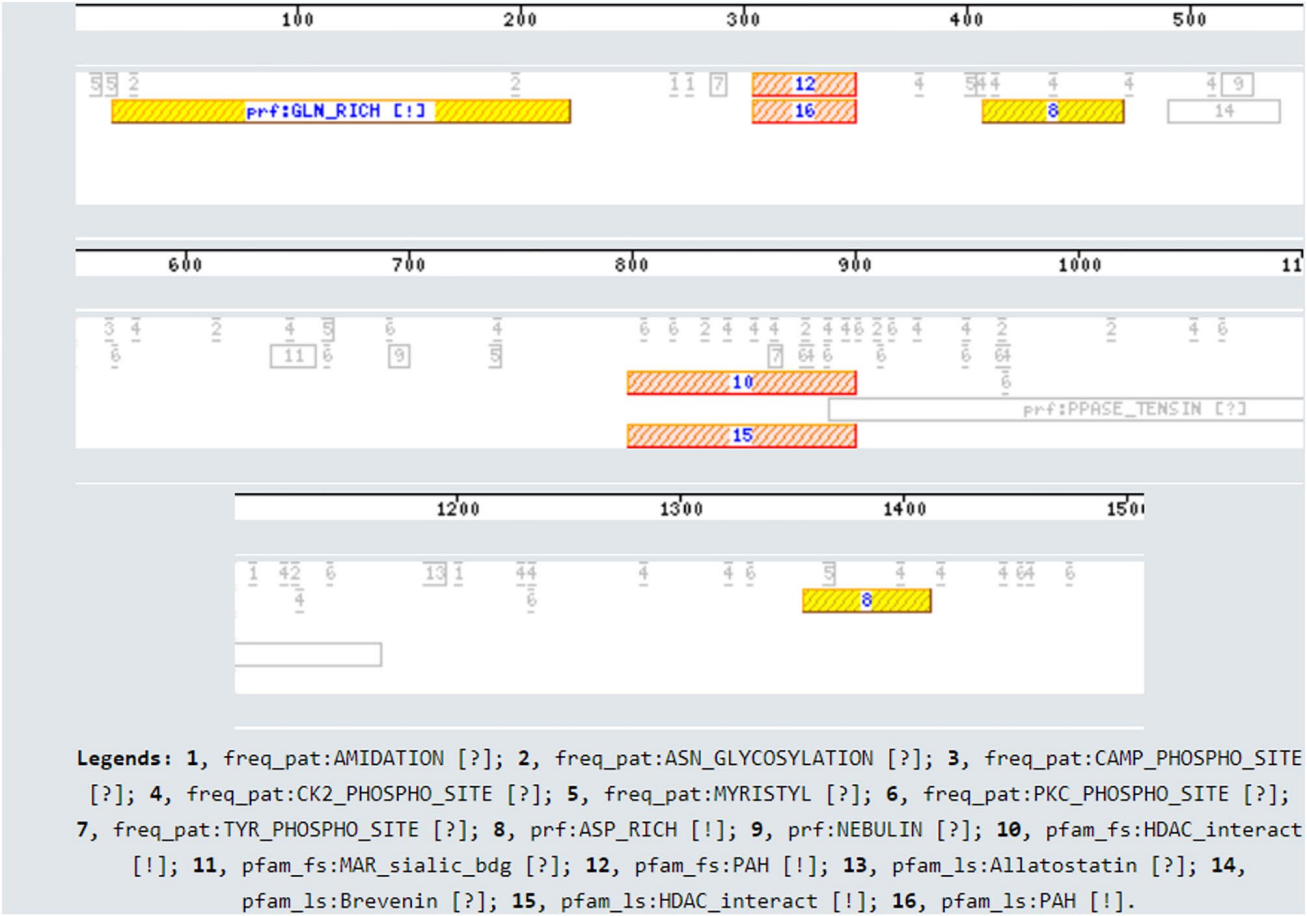
Schematic representation of motifs in *Caenorhabditis elegans* SIN-3 protein. Motif Scan^105^ was used for finding all the known motifs that occur in the protein sequence of SIN-3 protein using HAMAP profiles [hamap], PROSITE patterns [pat], PROSITE patterns (frequent match producers) [freq_pat], Pfam HMMs (global models) [pfam_ls], Pfam HMMs (local models) [pfam_fs], PROSITE profiles [prf], More profiles [pre]. The legends at the bottom of the figure are provided for the key to the numbering of the protein domains/motifs. [!] represents a strong match (low likelihood of a false positive) while [?] represents a questionable or weak match (additional biological evidences are required for true/false negative status).

The SIN3 interactome is well studied in yeast, human, and mouse^52,53^. Therefore, the protein–protein interactions of these eukaryotic systems can be extrapolated to *ceSIN3* ortholog with computational methods^54^. GeneMANIA was used to obtain protein–protein interactions of SIN3 in different eukaryotic systems. The database extracted 100 physical interactions, represented in the form of a network image (Fig. S4), of the SIN3 protein for each species included in the study. In the nematode SIN-3 interactome, we found several protein interactions conserved across evolution (Fig. 3).

21 proteins out of the 100 *C. elegans* SIN-3 interactors were found to be conserved in one or more SIN3 interactomes of other model organisms (Table 3). While the number of conserved protein interactions seems low, this could be due to the limitations of studying only 100 protein–protein interactions within the database used to generate the protein interactome and ambiguity in the available data. These interactors are involved in essential biological processes like transcriptional regulation, signal transduction, development and lifespan regulation^55^. Notably, *ceSIN3* protein also participates in many unique protein interactions (Table S1). Some of these interactions might be mediated by Glutamine-rich and Aspartate repeats present outside the protein's functional domains (PAH and HID) and via similar repeats occurring in SIN-3 interacting nematode proteins.

**Table 3:** *Caenorhabditis elegans* SIN-3 protein interactors conserved in SIN3 interactome of other model organisms (Cele, *Caenorhabditis elegans* Hsap, *Homo sapiens*; Mmus, *Mus musculus*; Drer, *Danio rerio*; Dmel, *Drosophila melanogaster*; Scer, *Saccharomyces cerevisiae*).

S. no.	Cele	Drer	Dmel	Hsap	Mmus	Scer
1	AKT-1, AKT-2, SGK-1					Sch9
2	CEH-27	Nkx2.2a		NKX2-2		
3	CEH-28			NKX3-2		
4	CEH-62		Bcd			
5	DCP-66	Gatad2ab	Simj		GATAD2A	
6	FOS-1					Sko1
7	HCF-1	Hcfc1b	Hcf	HCFC1		
8	HLH-3	Tal1			TAL1	
9	JMJD-3.2		Lid	KDM5A	KDM5B	Cyc8
10	KLF-2	Sp3a		KLF9, KLF16	SP3, KLF1	Swi5
11	MED-2				GATA4	
12	MXL-3	Mntb, Mxd1, Mxi1		MNT, MXD1, MXI1, MAX, MXD3	MNT, MXD1, MXD4, MXI1, MAX	
13	NHR-23			NR1D2	THRAB	
14	SIN-3	Sin3ab, Sin3b, Sin3aa	Sin3A	SIN3A, SIN3B	SIN3A, SIN3B	Sin3
15	SOX-3	Sox2		SOX2	SOX2	
16	TAB-1				NANOG	
17	TAG-97				ETV6	
18	UNC-130					Fkh2, Fkh1
19	ZTF-14				Gli1	

Missing data is a major problem in large-scale profiling experiments, and their adverse effect on the downstream analysis is beyond the capacity of simple computational methods^56^. To find reliable protein–protein interactions of *ceSIN3*, interactions with experimental evidence should be analyzed along with the GeneMANIA dataset^57^. Therefore, *ceSIN3* protein–protein interactions were manually curated from published data and represented in a network constructed using Cytoscape (Fig. S5).

Orthology of the nematode proteins in the SIN-3 interactome, obtained via GeneMANIA and literature based evidence method, was checked in other model organisms included in the analysis using the Orthofinder tool. It was found that many of the SIN-3 interactors found in *C. elegans* were not conserved in other organisms (Fig. S6). This contraction in protein orthogroups could be due to the lower sequence identity of nematode proteins with higher eukaryotic proteins. Further, SIN3 might be involved in multiple transient interactions to be able to regulate several pathways at a time. This poses a challenge in the in-vitro characterization of SIN3 protein–protein interactions. Some *C. elegans* proteins involved in the SIN-3 interaction network are quite expanded in the nematode proteome. These proteins (HDA-1, NHR-67, MAB-3, ODD-2, AKT-1, AKT-2, EGL-38, ELT-7, LIN-15A, FKH-6, FOZI-1 etc) are involved in important processes like sex differentiation, multicellular development, cell differentiation and cell signaling pathways, thereby enabling SIN-3 mediated regulation via protein–protein interaction.

It has been well-established that both SIN3 isoforms, SIN3A and SIN3B, form distinct protein complexes. Phylogenetic analysis has revealed that *ceSIN3* shares a closer relationship with SIN3A isoforms. Therefore, it was imperative to evaluate the nematode protein for conservation of SIN3A/SIN3B specific protein interactions in model organisms which harbor multiple isoforms of the SIN3 protein. While it was observed that very few SIN3A/SIN3B specific interactions are conserved in *ceSIN3*, no particular preference for SIN3A specific interactions is seen in humans, mice, or zebrafish. This might be due to the similarity in the protein sequence identity of the *ceSIN3* protein with SIN3A and SIN3B isoforms.

### Context-specific protein annotation

**Figure 4:**
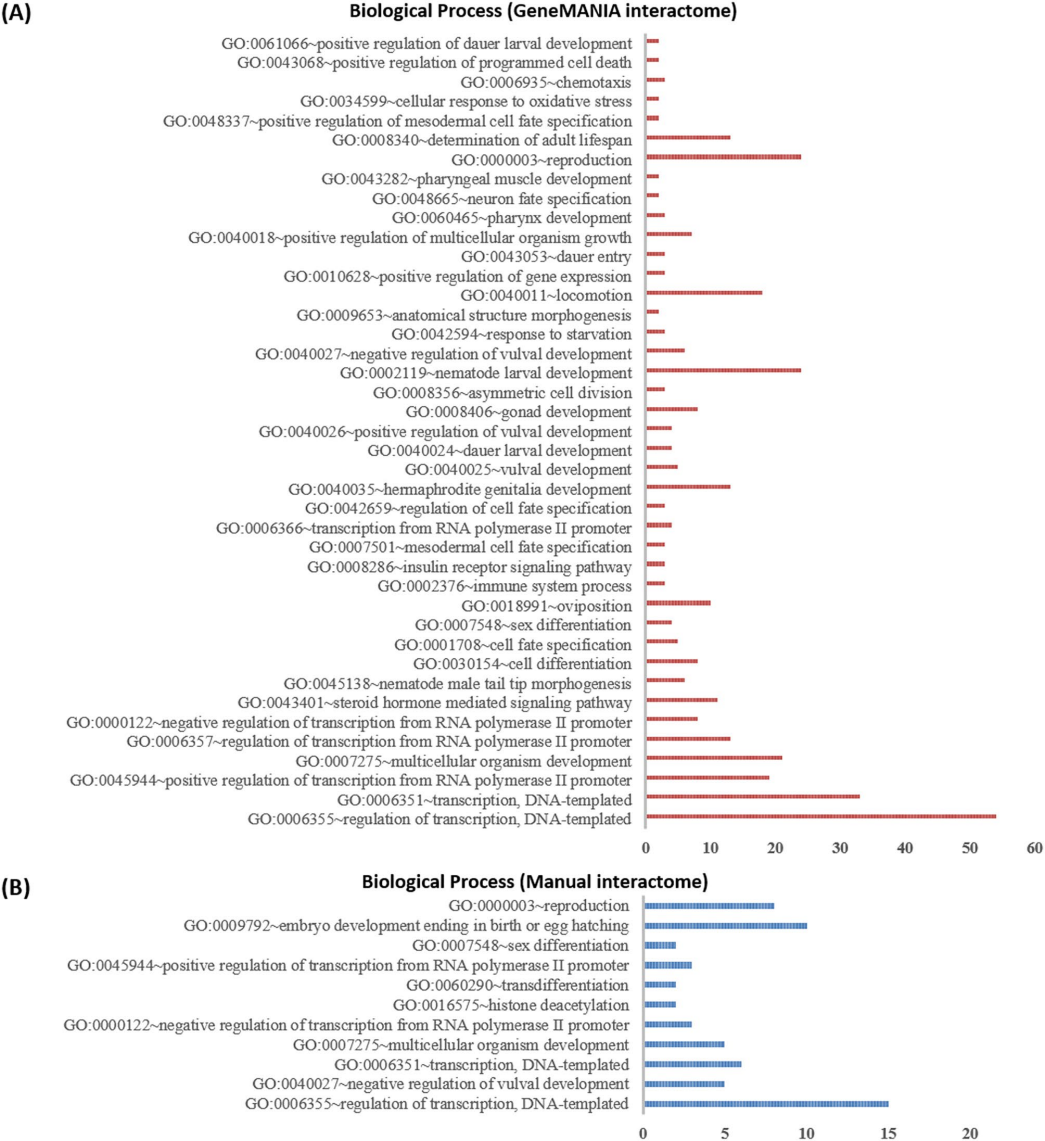
Biological processes associated with protein interactome of *C. elegans* SIN-3 protein, obtained from (**A**) GeneMANIA database and (**B**) Direct Evidence method using the NIH LHRI DAVID 6.8 service. In order to find reliable protein–protein interactions*,* experimental evidence was analyzed along with the GeneMANIA dataset via literature based evidence method. The y-axis represents the GO biological processes, while the x-axis represents the number of proteins associated with a particular biological process.

**Figure 5:**
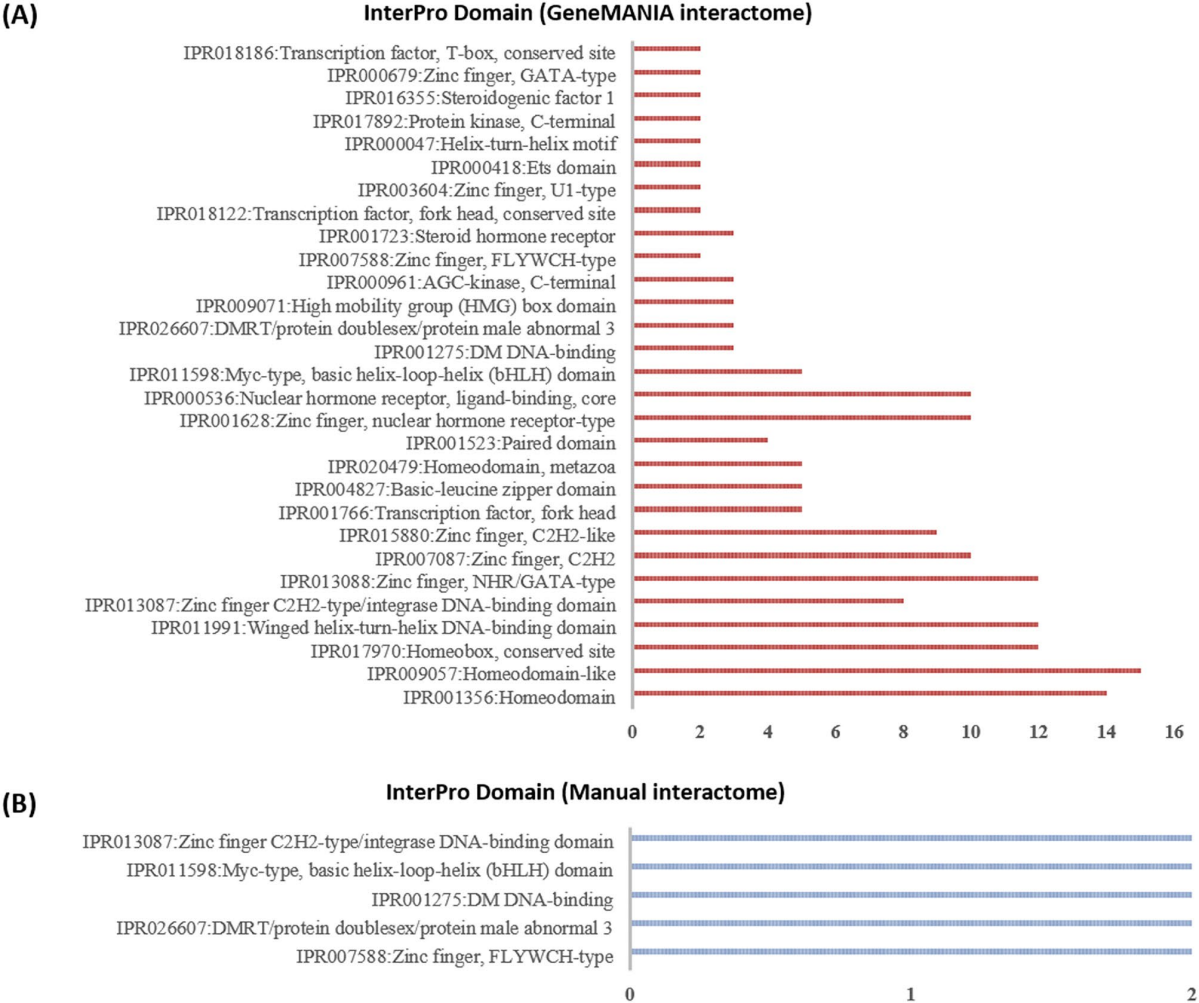
InterPro domain analysis of protein interactome of *C. elegans* SIN-3 protein, obtained from (**A**) GeneMANIA database and (**B**) direct evidence method using the NIH LHRI DAVID 6.8 service. The y-axis represents the InterPro domains, while the x-axis represents the number of proteins associated with a particular domain.

A protein can have multiple functions depending on the tissue/cellular context in which the protein is present. Some of the functions assigned to a protein are more relevant in certain conditions, such as programmed cell death, determination of adult lifespan and cellular response to oxidative stress. In this study, we investigated protein–protein functional associations by using GO terms, InterPro domains and KEGG pathways^29,58^ to identify the participation of SIN-3 protein interactors in apoptosis, autophagy and longevity. GO ‘biological process’ classification of *ceSIN3* protein interactome reveals that 53% interactors are associated with the regulation of transcription, the majority being transcription factors (Fig. 4). Furthermore, when annotated with InterPro domains, most of the interactor proteins indicated the presence of DNA binding domains, homeobox domain, zinc finger domain and winged helix-turn-helix DNA-binding domain (Fig. 5) that assist in transcriptional regulation^59,60^. Previous studies also supported this by establishing SIN3 interaction with many transcriptional factors^3,9^.

GO term analysis revealed that some *ceSIN3* protein interactors are involved in the positive regulation of programmed cell death, cellular response to oxidative stress and determination of adult lifespan in *C. elegans* (Table 4). HLH-3 and CES-2 are involved in the positive regulation of programmed cell death, while DAF-16 and BAR-1 are involved in the cellular response to oxidative stress. Further, 12% of SIN-3 interactors are involved in the determination of adult lifespan. Among these SIN-3 interactors, BAR-1, DAF-12, JUN-1, and PHA-4 undergo a significant transcriptional up regulation in case of sin-3 deletion (q value < 0.05, log2 Fold Change > 1)^61^. The rest of the proteins are also modulated upon sin-3 deletion but at a lower statistical significance (q value > 0.05).

**Table 4:** *C. elegans* SIN-3 protein interactors involved in apoptosis, autophagy and longevity.

S. no.	Name of protein	Function	References
1	HLH-3	Induction of programmed cell death, regulation of axon extension involved in axon guidance; regulation of oviposition	^62,63^
2	CES-2	Promotes programmed cell death and autophagy	^63–65^
3	DAF-16	Supports resistance to oxidative stress and longevity, apoptosis, defense response to other organism; regulation of dauer larval development; regulation of primary metabolic process	^66–72^
4	BAR-1	Life-span regulation, multicellular organism development; reproductive behavior; signal transduction	^73,74^
5	AKT-1	Induction of DNA damage-dependent apoptosis, inhibition of apoptosis, cellular protein modification process; determination of adult lifespan; signal transduction	^75–78^
6	AKT-2	Induction of DNA damage-dependent apoptosis, inhibition of apoptosis, cellular protein modification process; determination of adult lifespan; insulin receptor signaling pathway	^75–77^
7	SIR-2.1	Chromosome organization, determination of adult lifespan, induction of p53/cep-1 independent apoptosis, and autophagy	^79–82^
8	SGK-1	Determination of adult lifespan, mesendoderm development; peptidyl-serine phosphorylation; regulation of protein localization to basolateral plasma membrane	^83–86^
9	RLE-1	Determination of adult lifespan, response to heat; and ubiquitin-dependent protein catabolic process	^87,88^
10	FOS-1	Cell invasion, lifespan regulation	^89,90^
11	SET-1	Embryo development, histone methylation, apoptosis lifespan regulation	^91–94^
12	PHA-4	Multicellular organism development; determination of adult lifespan; positive regulation of cellular metabolic process; response to caloric restriction	^68,95,96^
13	HCF-1	Dauer exit; determination of adult lifespan	^97^
14	DAF-12	Determination of adult lifespan; heat acclimation; positive regulation of dauer larval development	^98–101^
15	JUN-1	Determination of adult lifespan; response to starvation	^102^

**Figure 6:**
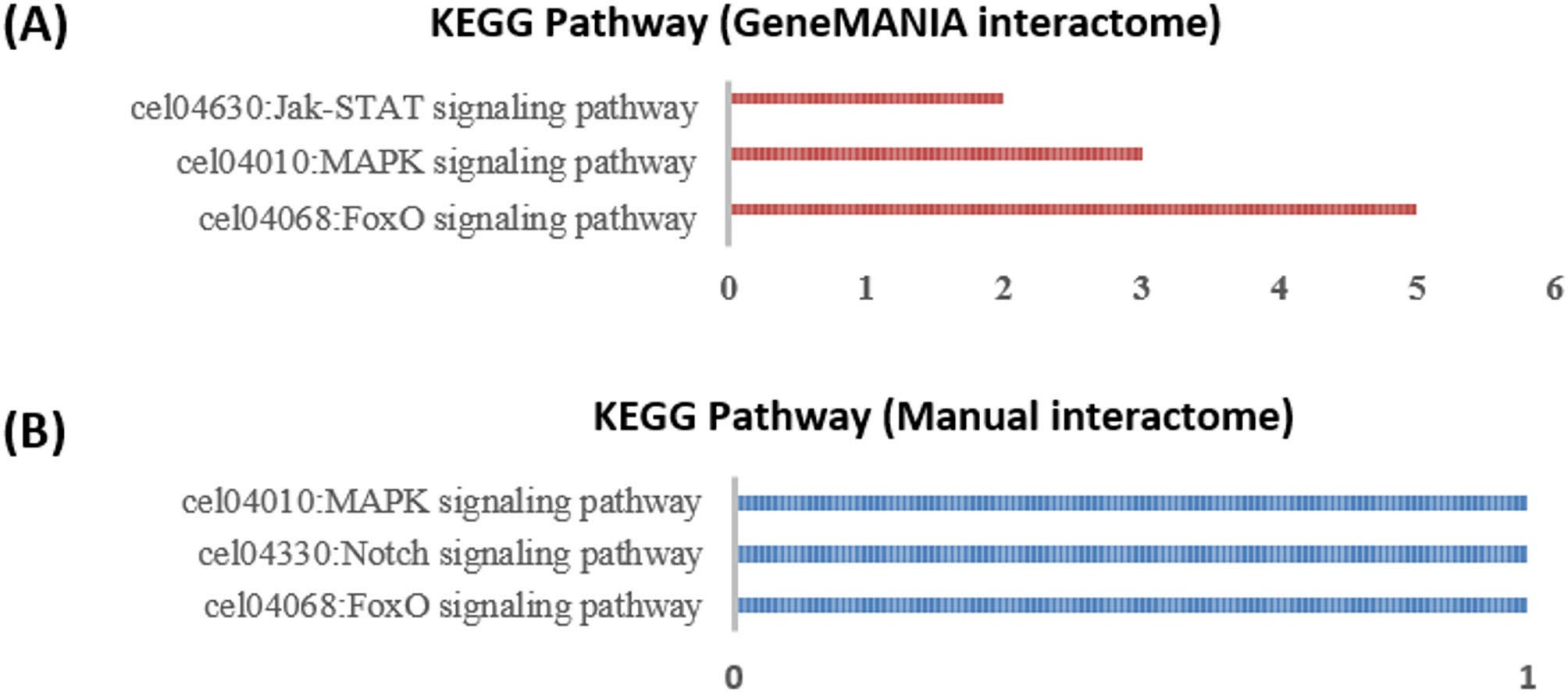
KEGG pathway analysis of protein interactome of *C. elegans* SIN-3 protein, obtained from (**A**) GeneMANIA database and (**B**) direct evidence method using the NIH LHRI DAVID 6.8 service. The y-axis represents the KEGG Pathways, while the x-axis represents the number of proteins associated with a particular KEGG Pathway.

Notable KEGG pathways corresponding to cell death and longevity were associated with some of these *ceSIN3* interactors. The FOXO (controls starvation-induced autophagy along with Foxk1/2 and SIN3 proteins), MAPK and Jak-STAT (Fig. 6) pathways involve DAF-16, SIR-2.1, AKT-1/2, SGK-1, MXL-3 from the SIN-3 interactome.

### PAH domain interaction motif in nematode protein interactors

Motifs are used as scaffolds by proteins and transcription factors interacting with SIN3 PAH domains. LxxLL (x indicates any amino acid) motif is an acidic leucine (L)–rich protein binding motif used by proteins to interact with the PAH1 domain of SIN3 protein^103^. SIN3 interactors like SAP25 and MAD1 interact with the PAH1 domain of SIN3 protein via this unique sequence^104^. Such motifs are likely to be conserved in the case of *ceSIN3* PAH domain protein interactions. This unique motif was checked for its presence in the SIN-3 interactor protein sequences. It was found that AKT-2, AKT-1, SIR-2.1, CES-2, and BAR-1, which are associated with apoptosis, autophagy, and longevity, harbor one or more LxxLL-type motifs (Table 5). Since the *ceSIN3* PAH domain is primarily similar to the PAH1 domain of other SIN3 homologs, *C. elegans* proteins might interact with the SIN-3 protein via the LxxLL motif. In addition, it was seen that six nematode proteins (DCP-66, FOZI-1, TAF-12, GEI-13, SMA-9, CEH-36) interacting with *ceSIN3* protein contain a stretch of Glutamine-rich repeats or Aspartate repeats (Table S3).

**Table 5:** *Caenorhabditis elegans* SIN-3 protein interactors containing LxxLL motifs. LxxLL motif is an acidic leucine (L)–rich protein binding motif used by proteins to interact with the PAH1 domain of SIN3 protein. The protein sequences were obtained from NCBI protein and Uniprot databases, and the motifs were scanned using the ScanProsite^106^ tool.

Gene name	Accession no.	No. of LxxLL motifs	Name of the LXXLL motif
*sin-3*	A5JYW9.1	2	LgkLL, LmtLL
*sir-2.1*	NP_001255484.1	1	LsdLL
*ceh-62*	Q09602.1	1	LrlLL
*f57c9.4*	NP_491459.1	1	LpnLL
*mab-3*	CAB16489.1	1	LasLL
*ztf-2*	CAA95789.1	1	LasLL
wbgene00017431	CCD69466.1	1	LryLL
*ets-5*	CCJ09391.1	1	LleLL
*nhr-43*	CDR32746.1	1	LenLL
*akt-1*	NP_001023646.1	2	LenLL, LtgLL
*ces-2*	CAB05032.1	1	LrfLL
*imb-3*	CCD67936.1	2	LetLL, LvcLL
*fkh-7*	CCD67108.1	1	LskLL
*nhr-67*	Q9XVV3.1	1	LnfLL
*lin-15a*	Q27365.1	2	LvaLL, LneLL
*t04h1.2*	CAB01578.1	1	LrtLL
*nurf-1*	CAA0059157.1	4	LvvLL, LyqLL, LleLL, LelLL
*bar-1*	AAC17424.1	6	LrdLL, LlmLL, LanLL, LksLL, LhkLL, LslLL
*eyg-1*	NP_496116.1	1	LslLL
*akt-2*	CAA20936.1	2	LenLL, LsgLL
*gei-13*	CAA80171.2	2	LaaLL, LnaLL
*ztf-11*	CAB05737.2	1	LmsLL

### Effect of mutation in protein interactors

**Figure 7:**
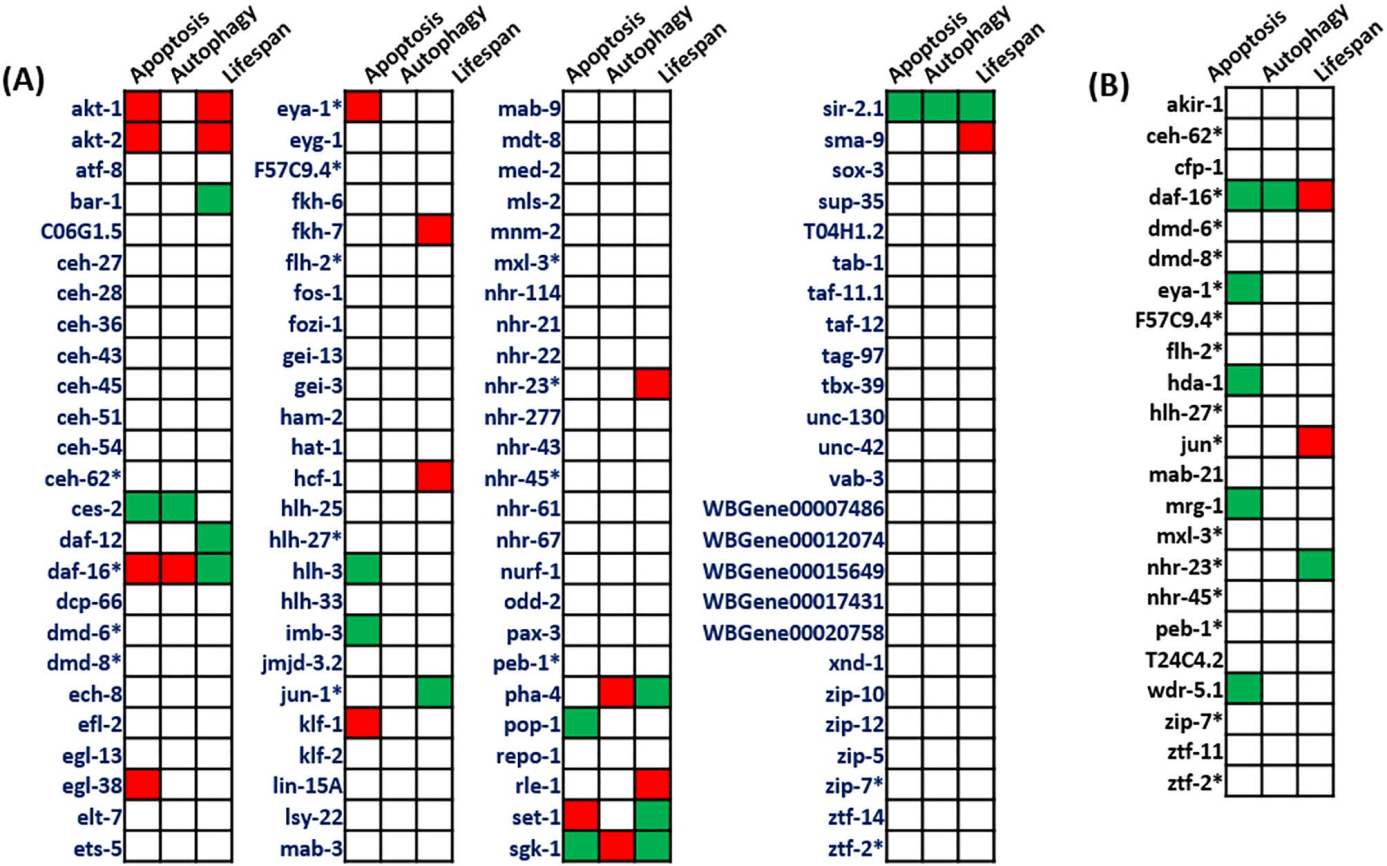
Analysis of SIN-3 protein interactome of mutants of *C. elegans* obtained from (**A**) GeneMANIA database and (**B**) direct evidence method using WormBase^107^ phenotype option. Green indicates an increase, while red indicates a decrease in apoptosis/autophagy/ longevity of *C. elegans* mutants.

Consequential mutant phenotypes provide evidence-based annotations for a majority of proteins. Such an approach helps to investigate the presence of SIN3 mutant-like phenotypes. Therefore, the mutant phenotypes associated with apoptosis, autophagy, and longevity were curated for all the proteins in the *ceSIN3* interactome (Fig. 7). We used extensive text mining to identify such phenotypes. We further wanted to check whether phenotypes associated with mutants of the interactor proteins were phenocopies known for the *sin-3* mutants. Mutant phenotypes of multiple proteins show *sin-3* mutant-like phenotypes with respect to apoptosis, autophagy, and longevity. Remarkably, four mutants, *pha-4* (ortholog of human FOXA1 and FOXA2), *daf-16* (ortholog of human FOXO1; FOXO3; and FOXO4), *sgk-1* (ortholog of human SGK2), and *sir-2.1* mutant (ortholog of human SIRT1), show elevated autophagy, programmed cell death and shortened lifespan like that of *sin-3* mutants. Other mutants such as *akt-2*, *akt-1*, *ces-2*, and *bar-1* also show phenotypes related to apoptosis and longevity. These interactor proteins seem to have a well-established connection with the apoptosis-autophagy-longevity axis (Konwar *et. al*., unpublished results)^13^.

## Conclusion

Our study identifies novel protein interactors of the SIN3 protein. The protein homology and phylogenetic relationship between the *ceSIN3* and other eukaryotic SIN3 isoforms provides a basis for the genetic and functional correlation of the *C. elegans* protein with the other homologs. Protein–protein interactions of *ceSIN3* protein revealed many interacting proteins associated with the regulation of programmed cell death, autophagy, and adult life span. Some of these interacting proteins, such as SIR-2.1, AKT-1/2, BAR-1, and CES-2, contain the SIN3 PAH domain interaction (LXXLL) motif, while others, like SMA-9, FOZI-1, and CEH-36, harbor Glutamine rich repeats known to be involved in protein–protein interactions. A few of these protein interactors also display mutant phenotypes similar to that of *sin-3* mutants, indicating their potential in regulating apoptosis, autophagy, and longevity. However, experimental evidence will provide better insight. It is important to check the protein interactions of the *ceSIN3* protein by performing a protein immunoprecipitation (IP) assay followed by Mass Spectroscopic (MS) analysis. This will confirm SIN3 protein interactions, which can be genetically studied for their role in apoptosis, autophagy, and longevity.

## Supplementary Information

Supplementary Information.

## Data Availability

All relevant data are within the manuscript and its Supporting Information file.
