# Supplementary material for: SIN-3 functions through multi-protein interaction to regulate apoptosis, autophagy, and longevity in Caenorhabditis elegans
Source: Sci Rep. 2022 Jun 22;12:10560. doi: 10.1038/s41598-022-13864-0 (PMC9217932; doi:10.1038/s41598-022-13864-0)
Supplement: Supplementary file 1 — Supplementary Information. [file 41598_2022_13864_MOESM1_ESM.pdf]

SIN-3 functions through multi-protein interaction to regulate apoptosis, autophagy, and longevity in *Caenorhabditis elegans*

Chandrika Konwar[1], Jayant Maini[3], Surbhi Kohli[1], Vani Brahmachari[1] and Daman Saluja[1,2*]

[1]Dr. B. R. Ambedkar Center for Biomedical Research, University of Delhi, Delhi-110007, India,

[2]Delhi School of Public Health, IoE, University of Delhi, Delhi-110007, India,

[3]Department of Biotechnology, Manav Rachna International Institute of Research and Studies, Surajkund Road, Faridabad – 121004, Haryana, India.

[*]To whom correspondence should be addressed.

Supplementary Figures

Fig. S1: Tabular representation of percentage sequence identity of (A) *C. elegans* SIN-3 protein and associated domains, (B) HID domain, (C) Sin3A_C domain and (D) PAH domain, with other model organisms (Hsap, *Homo sapiens*; Mmus, *Mus musculus*; Drer, *Danio rerio*; Dmel, *Drosophila melanogaster*; Scer, *Saccharomyces cerevisiae*).

(A)

**SIN3_Cele**	100	20.33	21.62	22.2	22	21.53	21.14	22.38	22.14	22.69
**SIN3_Scer**	20.33	100	28.58	30.51	30.49	29.37	29.55	29.12	29.33	29.04
**SIN3A_Dmel**	21.62	28.58	100	47.78	47.14	45.71	46.46	44.21	44.8	44.68
**SIN3A_Hsap**	22.2	30.51	47.78	100	98.11	78.03	76.18	56.52	54.35	54.25
**SIN3A_Mmus**	22	30.49	47.14	98.11	100	77.73	76.04	56.47	54.3	54.29
**SIN3AA_Drer**	21.53	29.37	45.71	78.03	77.73	100	78.74	54.22	53.21	52.87
**SIN3AB_Drer**	21.14	29.55	46.46	76.18	76.04	78.74	100	54.57	52.33	52.15
**SIN3B_Drer**	22.38	29.12	44.21	56.52	56.47	54.22	54.57	100	67.23	68.54
**SIN3B_Hsap**	22.14	29.33	44.8	54.35	54.3	53.21	52.33	67.23	100	91.26
**SIN3B_Mmus**	22.69	29.04	44.68	54.25	54.29	52.87	52.15	68.54	91.26	100

(B)

**SIN3_Cele_HID**	100	38.14	39.18	40.62	39.39	39.58	40.62	39.58	39.58	40.62
**SIN3_Scer_HID**	38.14	100	47.52	43	44	44	47	46	46	46
**SIN3A_Dmel_HID**	39.18	47.52	100	66.34	66.34	66.34	71.29	71.29	71.29	70.3
**SIN3B_Drer_HID**	40.62	43	66.34	100	95.05	95.05	86.14	86.14	86.14	82.18
**SIN3B_Hsap_HID**	39.39	44	66.34	95.05	100	100	86.14	84.16	84.16	82.18
**SIN3B_Mmus_HID**	39.58	44	66.34	95.05	100	100	86.14	84.16	84.16	82.18
**SIN3Aa_Drer_HID**	40.62	47	71.29	86.14	86.14	86.14	100	93.07	93.07	90.1
**SIN3A_Hsap_HID**	39.58	46	71.29	86.14	84.16	84.16	93.07	100	100	94.06
**SIN3A_Mmus_HID**	39.58	46	71.29	86.14	84.16	84.16	93.07	100	100	94.06
**SIN3Ab_Drer_HID**	40.62	46	70.3	82.18	82.18	82.18	90.1	94.06	94.06	100

(C)

**SIN3_Cele_Sin3a_C**	100	19.08	18.5	20.77	20.77	18.03	18.03	18.58	19.67	19.57
**SIN3_Scer_Sin3a_C**	19.08	100	22.68	23.68	23.31	21.05	25.19	23.31	22.93	25.28
**SIN3A_Dmel_Sin3a_C**	18.5	22.68	100	47.34	46.86	46.86	46.38	46.38	47.83	48.31
**SIN3A_Hsap_Sin3a_C**	20.77	23.68	47.34	100	97.67	77.78	76.92	52.17	53.18	57.24
**SIN3A_Mmus_Sin3a_C**	20.77	23.31	46.86	97.67	100	77.44	75.92	52.84	53.85	57.91
**SIN3AA_Drer_Sin3a_C**	18.03	21.05	46.86	77.78	77.44	100	79.12	49.49	50.17	53.9
**SIN3AB_Drer_Sin3a_C**	18.03	25.19	46.38	76.92	75.92	79.12	100	52.35	52.35	54.73
**SIN3B_Hsap_Sin3a_C**	18.58	23.31	46.38	52.17	52.84	49.49	52.35	100	93.33	74.41
**SIN3B_Mmus_Sin3a_C**	19.67	22.93	47.83	53.18	53.85	50.17	52.35	93.33	100	74.75
**SIN3B_Drer_Sin3a_C**	19.57	25.28	48.31	57.24	57.91	53.9	54.73	74.41	74.75	100

(D)

**SIN3_Cele_PAH**	100	55.56	48.89	53.33	53.33	53.33	53.33	54.55	51.11	51.11	31.11	33.33	31.11	31.11	33.33	29.55	31.11	31.11	31.11	27.91	26.67	28.89	28.89	28.89	28.89	28.89	28.89	28.89
**SIN3_Scer_PAH1**	55.56	100	75.56	75.56	75.56	75.56	75.56	75	68.89	68.89	33.33	28.89	31.11	31.11	26.67	29.55	35.56	35.56	28.89	23.26	24.44	26.67	26.67	24.44	24.44	24.44	24.44	22.22
**SIN3A_Dmel_PAH1**	48.89	75.56	100	77.78	77.78	77.78	77.78	75	68.89	68.89	28.89	26.67	31.11	31.11	28.89	31.82	33.33	33.33	31.11	25.58	24.44	28.89	28.89	26.67	26.67	26.67	26.67	24.44
**SIN3A_Hsap_PAH1**	53.33	75.56	77.78	100	100	100	100	84.09	80	80	31.11	28.89	31.11	31.11	35.56	34.09	33.33	33.33	35.56	20.93	28.89	26.67	26.67	24.44	24.44	26.67	26.67	24.44
**SIN3A_Mmus_PAH1**	53.33	75.56	77.78	100	100	100	100	84.09	80	80	31.11	28.89	31.11	31.11	35.56	34.09	33.33	33.33	35.56	20.93	28.89	26.67	26.67	24.44	24.44	26.67	26.67	24.44
**SIN3AA_Drer_PAH1**	53.33	75.56	77.78	100	100	100	100	84.09	80	80	31.11	28.89	31.11	31.11	35.56	34.09	33.33	33.33	35.56	20.93	28.89	26.67	26.67	24.44	24.44	26.67	26.67	24.44
**SIN3AB_Drer_PAH1**	53.33	75.56	77.78	100	100	100	100	84.09	80	80	31.11	28.89	31.11	31.11	35.56	34.09	33.33	33.33	35.56	20.93	28.89	26.67	26.67	24.44	24.44	26.67	26.67	24.44
**SIN3B_Drer_PAH1**	54.55	75	75	84.09	84.09	84.09	84.09	100	84.09	84.09	34.09	31.82	31.82	31.82	31.82	34.09	36.36	36.36	31.82	20.93	27.27	27.27	27.27	25	25	25	25	22.73
**SIN3B_Hsap_PAH1**	51.11	68.89	68.89	80	80	80	80	84.09	100	100	33.33	33.33	31.11	31.11	33.33	34.09	33.33	33.33	33.33	20.93	26.67	26.67	26.67	24.44	24.44	24.44	24.44	22.22
**SIN3B_Mmus_PAH1**	51.11	68.89	68.89	80	80	80	80	84.09	100	100	33.33	33.33	31.11	31.11	33.33	34.09	33.33	33.33	33.33	20.93	26.67	26.67	26.67	24.44	24.44	24.44	24.44	22.22
**SIN3_Scer_PAH2**	31.11	33.33	28.89	31.11	31.11	31.11	31.11	34.09	33.33	33.33	100	57.78	64.44	64.44	57.78	59.09	51.11	51.11	48.89	25.58	31.11	28.89	28.89	28.89	28.89	26.67	26.67	28.89
**SIN3A_Dmel_PAH2**	33.33	28.89	26.67	28.89	28.89	28.89	28.89	31.82	33.33	33.33	57.78	100	63.79	63.79	62.07	61.4	52.73	54.55	53.45	25.58	31.11	26.67	26.67	26.67	26.67	24.44	24.44	24.44
**SIN3A_Hsap_PAH2**	31.11	31.11	31.11	31.11	31.11	31.11	31.11	31.82	31.11	31.11	64.44	63.79	100	100	89.66	89.47	63.64	63.64	65.52	23.26	28.89	24.44	24.44	24.44	24.44	22.22	22.22	22.22
**SIN3A_Mmus_PAH2**	31.11	31.11	31.11	31.11	31.11	31.11	31.11	31.82	31.11	31.11	64.44	63.79	100	100	89.66	89.47	63.64	63.64	65.52	23.26	28.89	24.44	24.44	24.44	24.44	22.22	22.22	22.22
**SIN3AB_Drer_PAH2**	33.33	26.67	28.89	35.56	35.56	35.56	35.56	31.82	33.33	33.33	57.78	62.07	89.66	89.66	100	89.47	63.64	63.64	63.79	23.26	31.11	24.44	24.44	24.44	24.44	24.44	24.44	24.44
**SIN3AA_Drer_PAH2**	29.55	29.55	31.82	34.09	34.09	34.09	34.09	34.09	34.09	34.09	59.09	61.4	89.47	89.47	89.47	100	66.67	66.67	63.16	23.26	29.55	25	25	25	25	22.73	22.73	22.73
**SIN3B_Hsap_PAH2**	31.11	35.56	33.33	33.33	33.33	33.33	33.33	36.36	33.33	33.33	51.11	52.73	63.64	63.64	63.64	66.67	100	96.36	72.73	25.58	31.11	26.67	26.67	26.67	26.67	24.44	24.44	24.44
**SIN3B_Mmus_PAH2**	31.11	35.56	33.33	33.33	33.33	33.33	33.33	36.36	33.33	33.33	51.11	54.55	63.64	63.64	63.64	66.67	96.36	100	74.55	25.58	31.11	26.67	26.67	26.67	26.67	24.44	24.44	24.44
**SIN3B_Drer_PAH2**	31.11	28.89	31.11	35.56	35.56	35.56	35.56	31.82	33.33	33.33	48.89	53.45	65.52	65.52	63.79	63.16	72.73	74.55	100	30.23	31.11	26.67	26.67	26.67	26.67	26.67	26.67	24.44
**SIN3_Scer_PAH3**	27.91	23.26	25.58	20.93	20.93	20.93	20.93	20.93	20.93	20.93	25.58	25.58	23.26	23.26	23.26	23.26	25.58	25.58	30.23	100	32.56	41.86	41.86	44.19	44.19	34.88	34.88	34.88
**SIN3A_Dmel_PAH3**	26.67	24.44	24.44	28.89	28.89	28.89	28.89	27.27	26.67	26.67	31.11	31.11	28.89	28.89	31.11	29.55	31.11	31.11	31.11	32.56	100	64.44	64.44	64.44	64.44	68.89	68.89	66.67
**SIN3A_Hsap_PAH3**	28.89	26.67	28.89	26.67	26.67	26.67	26.67	27.27	26.67	26.67	28.89	26.67	24.44	24.44	24.44	25	26.67	26.67	26.67	41.86	64.44	100	100	88.89	93.33	73.33	73.33	73.33
**SIN3A_Mmus_PAH3**	28.89	26.67	28.89	26.67	26.67	26.67	26.67	27.27	26.67	26.67	28.89	26.67	24.44	24.44	24.44	25	26.67	26.67	26.67	41.86	64.44	100	100	88.89	93.33	73.33	73.33	73.33
**SIN3AA_Drer_PAH3**	28.89	24.44	26.67	24.44	24.44	24.44	24.44	25	24.44	24.44	28.89	26.67	24.44	24.44	24.44	25	26.67	26.67	26.67	44.19	64.44	88.89	88.89	100	93.33	66.67	66.67	68.89
**SIN3AB_Drer_PAH3**	28.89	24.44	26.67	24.44	24.44	24.44	24.44	25	24.44	24.44	28.89	26.67	24.44	24.44	24.44	25	26.67	26.67	26.67	44.19	64.44	93.33	93.33	93.33	100	68.89	68.89	73.33
**SIN3B_Hsap_PAH3**	28.89	24.44	26.67	26.67	26.67	26.67	26.67	25	24.44	24.44	26.67	24.44	22.22	22.22	24.44	22.73	24.44	24.44	26.67	34.88	68.89	73.33	73.33	66.67	68.89	100	100	88.89
**SIN3B_Mmus_PAH3**	28.89	24.44	26.67	26.67	26.67	26.67	26.67	25	24.44	24.44	26.67	24.44	22.22	22.22	24.44	22.73	24.44	24.44	26.67	34.88	68.89	73.33	73.33	66.67	68.89	100	100	88.89
**SIN3B_Drer_PAH3**	28.89	22.22	24.44	24.44	24.44	24.44	24.44	22.73	22.22	22.22	28.89	24.44	22.22	22.22	24.44	22.73	24.44	24.44	24.44	34.88	66.67	73.33	73.33	68.89	73.33	88.89	88.89	100

Fig. S2: Multiple sequence alignment of (A) *C. elegans* SIN-3 protein and associated domains, (B) PAH domain, (C) HID domain and (D) Sin3A_C domain, performed using L-INS-I method by MAFFT (v7.475). Hsap, *Homo sapiens*; Mmus, *Mus musculus*; Drer, *Danio rerio*; Dmel, *Drosophila melanogaster*; Scer, *Saccharomyces cerevisiae*; Cele, *C. elegans.*

(A) Multiple Sequence Alignment of SIN3

```
SIN3_CELE      1
MYN------------PPP---GG---------GGGNNGGDQSQQQPTNNATLFLLQMIQQSQHQQQHQNQQQQQLELQIRDQERILIEQQRMQH-----QQQQNQLLQGL
SIN3_SCER      1
MSQ----------------------------------------------------------------------------------------VWH------NSNSQSNDVA
SIN3A_DMEL     1
MMKRTRVDEVQFGTRPVPQTSGGVGVGVVGVTGGGPTSGGGGTATVGVNTTGVTIGTVVPSAHNATISGIGS-------------------IHHRILTPQHGGAQTIAYL
SIN3A_HSAP     1
MKRR--------------------------------------------------------------------------LDDQE---------------------------
SIN3A_MMUS     1
MKRR--------------------------------------------------------------------------LDDQE---------------------------
SIN3AA_DRER    1
MRRR--------------------------------------------------------------------------LEDQE---------------------------
SIN3AB_DRER    1
MKRR--------------------------------------------------------------------------LEDQD---------------------------
SIN3B_HSAP     1
MAHA-----------------GG---------GSG---------------------------------------------------------------------------
SIN3B_MMUS     1
MAHA----------------------------------------------------------------------------------------------------------
SIN3B_DRER     1
MAK-----------------------------------------------------------------------------------------------------------


SIN3_CELE     82
NQFPFNPLGLF-QVQAAVQAAQAQFAQNAQGSPIPFHIGSPLQPSH------SPAASAL--QQQYLL---PSHSP-AITPFARNSEAARNIEQFIAQEEAANVPRANSQQ
SIN3_SCER     17
TSNDATGSNERNEKEPSLQGNKPGFVQQQQRITLPS--LSALSTKE-EDRRDSNGQQALTSHAAHILGYPPPHSN-AMPSIATDSALKQPHEYHPRPKSSSSSPSINASL
SIN3A_DMEL    92
PSTTPTATNLKTTTSIVDSTTAGGPVGAGAQVAVGI--GSAAGGRSVVVSTGSTGTQTLQYTTSYSVASIQAGGT-LKANTADGANTVQIHVTGRRTANNPASAQTVSSS
SIN3A_HSAP    10
---------------------SPVYAAQQRRIP-----GSTEAFP----------------HQHRVLAPAPPVYE-AVSETM---------------QSATGIQYSVTPS
SIN3A_MMUS    10
---------------------SPVYAAQQRRIP-----GSTEAFS----------------HQHRVLAPAPPVYE-AVSETM---------------QSATGIQYSVAPN
SIN3AA_DRER   10
----------------------PVFTPQQRRLP-----GSTEGF------------------QHRVLAPAPAVFE-AVADSM---------------QPTPGIQYPLPQA
SIN3AB_DRER   10
-----------------------TVIGSQRRLP-----GGVDSF------------------QHRVLNSASSLFETAAPETM---------------QPSSSMQYSVSQT
SIN3B_HSAP    10
--------------------------------------GS----------------------------------------------------------------------
SIN3B_MMUS     5
--------------------------------------------------------------------------------------------------------------
SIN3B_DRER     4
--------------------------------------------------------------------------------------------------------------
```

```
SIN3_CELE    179
QSPLIRPIPQQQALNIQNLTSTQQAQQILAHHRQVPVQQVQHQQHIPTPPLALPIAQQGPISNEVPSVPPVVPATSAGCPQREPRQQQGGRRQNRPGRRK-----KPEGP
SIN3_SCER    123
MNAGPAPLP----------TVGAASFSLSRFDNPLPIKAPVHTEE----PKSYNGLQEEEKATQRPQDCKEVPAGVQPADAPDPSSNHADANDDNNNNENSHDEDADYRP
SIN3A_DMEL   199
SQTGTIRQR----------TISGTQTVATAVGNLATISQ-------------QQPVQQSPLGKA-QTPPSSVVANSIPVGGTTPPQGQSG----------------NATPR
SIN3A_HSAP   62
YQVSAMPQS----------SGSHG-PAIAAVH---------------------SSHHH-PTAVQ-PHGGQVVQSHAHPAPPVAPVQGQ-----------------QQFQR
SIN3A_MMUS   62
YQVSAVPQS----------SGSHG-PAIAAVH---------------------SSHHH-PTAVQ-PHGGQVVQSHAHPAPPVAPVQGQ-----------------QQFQR
SIN3AA_DRER  59
YQVSTVAQS----------SSGHSHTPSPAVH--------------------SGPHHHGPAAAQ-AHAPPPVQGHAHPPTPSASTQGQ-----------------QQFQR
SIN3AB_DRER  59
YQLS-MPQN----------SGGHGHTSSSAVH--------------------TGPHHHGP-AVQ-----PVGQAHSHATPAPAPTLGQ-----------------QQFQR
SIN3B_HSAP   12
------------------------------------------------------------------GAGGPAGRGLSGARWGRSGSAG------------------HEKLP
SIN3B_MMUS    5
------------------------------------------------------------------GSGGSAGRGFGGSRWGRSGSGG------------------HEKLP
SIN3B_DRER    4
------------------------------------------------------------------------IQAHSSTAKQINQIQDKAYVVQK-------QVQQQHFQK

SIN3_CELE    284
PRVDEALAYLRVIKSTFSSDVPVYHRFLEIMKDFRAQRIETPDVIEQVAELLYDSPELVLGFNTFLPTGYRITLTPDRK------YVFSSPQMQPR--------------
SIN3_SCER    219
LNVKDALSYLEQVKFQFSSRPDIYNLFLDIMKDFKSQAIDTPGVIERVSTLFRGYPILIQGFNTFLPQGYRIECSSNPD---DP-IRVTTP-------MGTTTVNNNISP
SIN3A_DMEL   270
LKVEDALSYLDQVKYQYADQPQIYNNFLDIMKEFKSHCIDTPGVIERVSTLFKGHTELIYGFNMFLPPGYKIEIHSDALGCSVPVVSMPSPPGAPTSTGTVHMLTGNSSM
SIN3A_HSAP   121
LKVEDALSYLDQVKLQFGSQPQVYNDFLDIMKEFKSQSIDTPGVISRVSQLFKGHPDLIMGFNTFLPPGYKIEVQTNDM------VNVTTP-------GQVHQIPTH---
SIN3A_MMUS   121
LKVEDALSYLDQVKLQFGSQPQVYNDFLDIMKEFKSQSIDTPGVISRVSQLFKGHPDLIMGFNTFLPPGYKIEVQTNDM------VNVTTP-------GQVHQIPTH---
SIN3AA_DRER  121
LKVEDALSYLDQVKLQFGNQPQVYNDFLDIMKEFKSQSIDTPGVISRVSQLFKGHPDLIMGFNTFLPPGYKIEVQTNDL------VNVTTP-------GQIHHITPH---
SIN3AB_DRER  115
LKVEDALSYLDQVKLQFGNQPQVYNDFLDIMKEFKSQSIDTPGVISRVSQLFKGHPDLIMGFNTFLPPGYKIEVQTNDL------VNVTTP-------GQIHHITPH---
SIN3B_HSAP   39
VHVEDALTYLDQVKIRFGSDPATYNGFLEIMKEFKSQSIDTPGVIRRVSQLFHEHPDLIVGFNAFLPLGYRIDIPKNGK------LNIQSP-------LTSQENSHN---
SIN3B_MMUS   32
VHVEDALTYLDQVKIRFGSDPATYNGFLEIMKEFKSQSIDTPGVIRRVSQLFHEHPDLIVGFNAFLPLGYRIDIPKNGK------LNIQSP-------LSSQDNSHS---
SIN3B_DRER   36
LKVEDALSYLDQVKIRFGNDPGIYNKFLDIMKEFKSQSIDTPGVINRVSQLFHGHPDLVLGFNAFLPPGYRIEIPKNGM------AFLQSP-------FSSQVSPGA---

SIN3_CELE    374
-------------VLLSPDERRARAIEAGA----QAVGAIELGSQEGIS---------KDEDRTIEDED---------------------------MDKSKEKDDVDGI
SIN3_SCER    318
S-----GRGTTDAQELGSFP--------------ESDG-------NGVQ---------QPSNVPMVPSS---------------------------VYQSEQNQDQQQS
SIN3A_DMEL   380
SGAGHIAIKTTNAATLTPAAGAGAAAAAAAVAQIQSAGAVNLMTHGGASLTQTTIHALQQATPPQSQSPGGGHVHVSVTAANAVVPGQPGISVSAHNVPQNYSRDRERAT
SIN3A_HSAP   215
------GIQPQP----QPPP--------------QHPS--------------------QPSAQSAPA--------------PAQPAPQP--------PPAKVSKPSQLQA
SIN3A_MMUS   215
------GIQPQP----QPPP--------------QHPS--------------------QPSSQSAPT--------------PAQPAPQP--------TAAKVSKPSQLQA
SIN3AA_DRER  215
------GISVQNIPIVPPPP--------------PSQ---------------------QQNQITTNPPN-----------LATTPPAQP--------APK--TKPLQSPV
SIN3AB_DRER  209
------GISVQNIPITQPAP--------------HQP---------------------QPAPPTSTA--------------ALVIPSQP--------TPAKISKPMQSPA
SIN3B_HSAP   133
----H-GDGAED-----------------------------------------------------------------------------------------------
SIN3B_MMUS   126
----H-GDCGED-----------------------------------------------------------------------------------------------
SIN3B_DRER   130
------GRSTGSSVVSASSS-----------AVVEAAGP------------------AQNEAVTSPES---------------------------IASSSGPPEQSSK

SIN3_CELE    430
D-DEDD----------------EESGIEDKNNEEMME---------------------EDNHLIEEIICDDRKKDDCEDSQQEIEMSSELAAHTLNIIELLKKSFLARPT
SIN3_SCER    365
L-PLLA----------------TSSGLPSIQQPEMPA----------------HRQIPQSQSLVP---------QEDAKKNVDVEFS-----QAISYVNKIKTRFADQPD
SIN3A_DMEL   490
ITPTGQMAGAAANVNASASIVVGGPPTPNSLSELSPHGGAGGGPGAGAAQHNLHHIQQAHQSILL---------GETGQQNQPVEFN-----HAITYVNKIKNRFQNQPA
SIN3A_HSAP   259
HTPASQ----------------QTPPLPPYASPRSPP---------------VQPHTPVTISLGT---------APSLQNNQPVEFN-----HAINYVNKIKNRFQGQPD
SIN3A_MMUS   259
HTPASQ----------------QTPPLPPYASPRSPP---------------VQPHTPVTISLGT---------APSLQNNQPVEFN-----HAINYVNKIKNRFQGQPD
SIN3AA_DRER  263
LTPSSQ----------------PNPSIPPYASPRSPP---------------LQPNTPVSSMPI----------APPLQNNQPVEFN-----HAINYVNKIKNRFQGQPN
SIN3AB_DRER  256
LTPTSQ----------------PNPSIPSYASPRSPS---------------VQSHTPISSSTAG---------STPLQNNQPVEFN-----HAINYVNKIKNRFQGQPE
SIN3B_HSAP   140
----------------------FKQQVPYKEDKPQVP-----------------------------------------LESDSVEFN-----NAISYVNKIKTRFLDHPE
SIN3B_MMUS   133
----------------------FK-QMSYKEDRGQVP-----------------------------------------LESDSVEFN-----NAISYVNKIKTRFLDHPE
SIN3B_DRER   176
L---------------------SLPLPNRESQSQPA----------------------ATSV-----------SPPTSEPSPVEFD-----SAISYVNKIKNRFLDNPE

SIN3_CELE    502
KLVDFMTFIDFFMSDQQYKKDMEKLRKDDEDDEIEENEKIEVDDVPGPSNAPQEIKKPDDIEKKDSSKNLQIEESCSDYLVSMLANCCIGEPDLLAATIDFLPYLGKLLV
SIN3_SCER    428
IYKHFLEILQTYQREQKPI----------------------------------NEV-------------------------YAQVTHLFQNAPDLLEDFKKFLPDSSASAN
SIN3A_DMEL   586
```

```
KYKKFLEILHDYQREQKVMKE--------------------GSLNQGKMLTEQEV-------------------------YTQVAKLFGQDEDLLREFGQFLPDATNH--
SIN3A_HSAP   324
IYKAFLEILHTYQKEQRNAKE--------------------AGGNYTPALTEQEV-------------------------YAQVARLFKNQEDLLSEFGQFLPDANSSVL
SIN3A_MMUS   324
IYKAFLEILHTYQKEQRNAKE--------------------AGGNYTPALTEQEV-------------------------YAQVARLFKNQEDLLSEFGQFLPDANSSVL
SIN3AA_DRER  327
IYKSFLEILHKYQKEQRNAKE--------------------AGGSYTPVLTEQEV-------------------------YAEVAQLFKNQEDLLSEFGQFLPDANSTML
SIN3AB_DRER  321
VYKSFLEILHTYQKEQRNAKE--------------------AGGNYTPTLTEQQV-------------------------YAQVAQLFKNQEDLLSEFGQFLPDANSSVL
SIN3B_HSAP   182
IYRSFLEILHTYQKEQLNTR-----------------------GRPFRGMSEEEV-------------------------FTEVANLFRGQEDLLSEFGQFLPEAKRSLF
SIN3B_MMUS   174
IYRSFLEILHTYQKEQLHTK-----------------------GRPFRGMSEEEV-------------------------FTEVANLFRGQEDLLSEFGQFLPEAKRSLF
SIN3B_DRER   226
TYRAFLEILHTYQKEQLEVKE--------------------SRGRSTGGMTEDEV-------------------------FSKVASLFKGQEDLLAEFGQFLPDAKRSLF

SIN3_CELE    612
NGSDAIALKIKTILHFSATNDRN--------------------------------DIPPV-------------------------NRV----------------------
SIN3_SCER    480
QQVQHAQQ------HAQQQHEAQ--------------MHAQAQA--------QAQAQAQVEQ-----------------------QKQ-QQQFLYPASG--------YYG
SIN3A_DMEL   649
QSGQYMSK------SASVHNDHGKRPTATLSGGAHITMSSASPAPSGSPLHLGATTLPQIDKSAHAAAIGNLSAVNTSVSIKTYNNNQ-QQQNHVIGSGLNATRNDILFE
SIN3A_HSAP   389
LSKTTAEK------VDSVRNDHG-----------------------------GTVKKPQL-------------------------NNKPQR-------------------
SIN3A_MMUS   389
LSKTTAEK------VDSVRNDHG-----------------------------GTVKKPQL-------------------------NNKPQR-------------------
SIN3AA_DRER  392
LGKTTAEK------AESVRNDHG-----------------------------GTVKKPQL-------------------------NNK-QR-------------------
SIN3AB_DRER  386
LSKTTAEK------AEAVRIDHG-----------------------------GTVKRPQL-------------------------NNK-QR-------------------
SIN3B_HSAP   244
TGNGPCEM------HSVQKNEHD--------------------------------KTPEH-------------------------SRK----------------------
SIN3B_MMUS   236
TGNGSCEM------NSGQKNE-E--------------------------------KSLEH-------------------------NKK----------------------
SIN3B_DRER   291
TG-GSLPL------KKVEEEELN-----------------------------------KQ-------------------------TKK----------------------

SIN3_CELE    643
------------------------------------------------------------------NPSD----------------------------------------
SIN3_SCER    530
HPSNRGIPQQ---------------NLPPIGSFSPPTNGSTVHEAYQDQQHMQPPHFMPLPSIVQHGPNM----VHQGIANENPPLSDL-------------RTSLTEQY
SIN3A_DMEL   752
KDYHAGLQQQAHQRGAGVGGHHHLAGTAAGANIGRPGVGASVMVSY-DKEHRNNHH---VQKYVGHAPNQNLTHGHNAKKSPSYGIPSVIGSMPHISDNSLDRSSPGISY
SIN3A_HSAP   420
-------------------------------------------------------------------PSQN---GCQIRRHP-TGTT-----------------------
SIN3A_MMUS   420
-------------------------------------------------------------------PSQN---GCQIRRHSGTGAT-----------------------
SIN3AA_DRER  422
-------------------------------------------------------------------PNQN---GCQIRKHSTTAIT-----------------------
SIN3AB_DRER  416
-------------------------------------------------------------------FNQN---GCQIRRHSGPPTT-----------------------
SIN3B_HSAP   269
----------------------------------------------------------------------------RSRPSLLRPVS-----------------------
SIN3B_MMUS   260
----------------------------------------------------------------------------RSRPSLLRPVS-----------------------
SIN3B_DRER   312
----------------------------------------------------------------------------RPRPMLLPHMT-----------------------

SIN3_CELE    647
-----------------------VDMDL---VKQMEKCKMGTKKNEKL---KLKVAGQGDEGATVELMILKKSYRILYERLKSRTTPNQLSHLMVLINAYANLDITREQL
SIN3_SCER    608
APSSIQHQQQHPQSISPIANTQYGDIPVRPEIDLDPSIVPV--------VPEPTEPIENNISLNEEVTFFEKAKRY----IGNKHLYTEFLKILNLYS---------QDI
SIN3A_DMEL   858
ATPPLP-SGPHGQHNSGSATRRPGDDSL---VGHYASGAPPAKRPKPY-CRDVSFSEASSKCTISDAAFFDKVRKA----LRSPEVYDNFLRCLTLFN---------QEI
SIN3A_HSAP   436
--------------------------------------PPVKKKPKLLNLKDSSMADASKHGGGTESLFFDKVRKA----LRSAEAYENFLRCLVIFN---------QEV
SIN3A_MMUS   437
--------------------------------------PPVKKKPKLMSLKESSMADASKHGVGTESLFFDKVRKA----LRSAEAYENFLRCLVIFN---------QEV
SIN3AA_DRER  439
--------------------------------------PPVKKKLKLMNIKDSSGAEAGKHGGGTESLFFEKVRKV----LRSAEAYDNFLRCLVIFN---------EEI
SIN3AB_DRER  433
--------------------------------------PPVKKKQKFA-VKDPSVAEASKLGIGAESLFFEKVRKA----LRSSEAYDNFLRCLVIFN---------QEV
SIN3B_HSAP   280
--------------------------------------APAKKKMKLRGTKDLSIAAVGKYGTLQEFSFFDKVRRV----LKSQEVYENFLRCIALFN---------QEL
SIN3B_MMUS   271
--------------------------------------APAKKKMKLRGTKDLSIAAVGKYGTLQEFSFFDKVRRV----LKSQEVYENFLRCIALFN---------QEL
SIN3B_DRER   323
--------------------------------------PLLKKKMKYSCSKDPSFASVGKHGVLREFTFFDKVRRL----LKSQEVYENFLRCIALFN---------QEV

SIN3_CELE    728
ISELPKIMGTSGSDLEMIILQLLGAEKEPKNRPENDMD-----------AVM-----------RKDLPAIQPKRGLRDQKMLQQVKN---VEAATVCTLGPSYRFM-KDT
SIN3_SCER    697
L---------DLDDLVEKVDFYLGSNKELFTWFKNFVG-------------------------YQE----------KTKCIENIVHEKHRLDLDLCEAFGPSYKRLPKSD
SIN3A_DMEL   950
V---------SKTELLGLVSPFLMKFPDLLRWFTDFLGPPSGQPAGGLIDGMPLAATQRQGGGSSNSSHDRG--TSHQSAAEYV-QD---VDLSSCKRLGASYCALPQST
SIN3A_HSAP   495
I---------SRAELVQLVSPFLGKFPELFNWFKNFLG-------------------------YKESVHLET--YPKERATEGIAME---IDYASCKRLGSSYRALPKSY
SIN3A_MMUS   496
I---------SRAELVQLVSPFLGKFPELFNWFKNFLG-------------------------YKESVHLES--FPKERATEGIAME---IDYASCKRLGSSYRALPKSY
SIN3AA_DRER  498
```

```
I---------SRAELVQLVVPFLGKFPELFTWFKNFLG-------------------------YKEMSHLET--YPKERATEGIAME---IDYASCKRLGSSYRALPKSY
SIN3AB_DRER   491
I---------SRAELVQLVLPFLGKFPELFTWFKNFLG-------------------------YRECAHIES--FPKERATEGIAME---IDYASCKRLGSSYRALPKSF
SIN3B_HSAP    339
V---------SGSELLQLVSPFLGKFPELFAQFKSFLG-------------------------VKELSFAPP---MSDRSGDGISRE---IDYASCKRIGSSYRALPKTY
SIN3B_MMUS    330
V---------SGSELLQLVSPFLGKFPELFAQFKSFLG-------------------------VKELSFAPP---MSDRSGDGISRE---IDYASCKRIGSSYRALPKTY
SIN3B_DRER    382
V---------SGAELLQLVTPFLGKFPELYTQFKSFLG-------------------------DKELSHVIS--GLSDRYMEGGGRE---VDYASCKRLGSSYRALPKTY

SIN3_CELE     812
KATDCSGRVELDDDLK-----------------------------GVLNDTWTSIPSWSSEDTGSQAIKKSNLEEFHFKTEDERYELDIIVDSNRTVIEQLSKTLRDYEA
SIN3_SCER     763
TFMPCSGRDDMCWE--------------------------------VLNDEWVGHPVWASEDSGFIAHRKNQYEETLFKIEEERHEYDFYIESNLRTIQCLETIVNKIEN
SIN3A_DMEL  1045
VPKKCSGRTALCRE--------------------------------VLNDKWVSFPTWASEDSTFVTSRKTQFEETIYRTEDERFELDLVIEVNSATIRVLENLQKKMSR
SIN3A_HSAP    566
QQPKCTGRTPLCKE--------------------------------VLNDTWVSFPSW-SEDSTFVSSKKTQYEEHIYRCEDERFELDVVLETNLATIRVLEAIQKKLSR
SIN3A_MMUS    567
QQPKCTGRTPLCKE--------------------------------VLNDTWVSFPSW-SEDSTFVSSKKTQYEEHIYRCEDERFELDVVLETNLATIRVLEAIQKKLSR
SIN3AA_DRER   569
QLPKCTGRTAICKE--------------------------------VLNDTWVSFPSW-SEDSTFVSSKKTQYEEHMYRCEDERFELDVVLETNLATIRVLETTQKKLSR
SIN3AB_DRER   562
QQPRCTGRTPLCKE--------------------------------VLNDTWVSFPSW-SEDSTFVSSKKTQYEEHIYRCEDERFELDVVLETNLATIRVLEAVQKRISR
SIN3B_HSAP    409
QQPKCSGRTAICKELDHWTLLQGSWTDDYCMSKFKNTCWIPGYSAGVLNDTWVSFPSW-SEDSTFVSSKKTPYEEQLHRCEDERFELDVVLETNLATIRVLESVQKKLSR
SIN3B_MMUS    400
QQPKCSGRTAICKE--------------------------------VLNDTWVSFPSW-SEDSTFVSSKKTPYEEQLHRCEDERFELDVVLETNLATIRVLESVQKKLSR
SIN3B_DRER    453
QQPKCSGRTAICKE--------------------------------VLNDTWVSFPSW-SEDSTFVSSKKTPYEEQLHRCEDERFELDVVLETNLATIRVLESVQKKLSR

SIN3_CELE     893
MSDEDKKSFKLDKWLNASSRSTTIRVLAKVFT-NSAQDFIDAAQKNPLVGLRRILESLKEKDLLWSRFQQDTNRTWRDALDKQMSAATTILNNQHKNYDQKAFKSKPLVN
SIN3_SCER     841
MTENEKANFKLPPGLGHTSMTIYKKVIRKVYDKERGFEIIDALHEHPAVTAPVVLKRLKQKDEEWRRAQREWNKVWRELEQKVFFKSLDHLGLTFKQADKKLLTTKQLIS
SIN3A_DMEL  1123
MSTEELSKFHLDDHLGGTSQTIHQRAIHRIYG-DKSGEIITGMKKNPFVAVPIVLKRLKVKEEEWREAQKTFNKQWREQNEKYYLKSLDHQAINFKPNDMKALRSKSLFN
SIN3A_HSAP    643
LSAEEQAKFRLDNTLGGTSEVIHRKALQRIYA-DKAADIIDGLRKNPSIAVPIVLKRLKMKEEEWREAQRGFNKVWREQNEKYYLKSLDHQGINFKQNDTKVLRSKSLLN
SIN3A_MMUS    644
LSAEEQAKFRLDNTLGGTSEVIHRKALQRIYA-DKAADIIDGLRKNPSIAVPIVLKRLKMKEEEWREAQRGFNKVWREQNEKYYLKSLDHQGINFKQNDTKVLRSKSLLN
SIN3AA_DRER   646
MSAEEQAKFRLDNTLGGFSEIIHRKAIQRIYG-DKAPDIIDGLKKNPAASVPIVLKRLKLKEEEWREAQRGFNKIWREQNEKYYLKSLDHQGINFKQNDTKVLRSKTLLN
SIN3AB_DRER   639
MSAEEQAKFRLDNTIGGSSEVIHRKAIQRIYG-DKAPDIIDGLKTNPAVSVPIVLKRLKIKEEEWREAQRGFNKIWREQNEKYYLKSLDHQGINFKQNDTKVFRSKTLLN
SIN3B_HSAP    518
MAPEDQEKFRLDDSLGGTSEVIQRRAIYRIYG-DKAPEIIESLKKNPVTAVPVVLKRLKAKEEEWREAQQGFNKIWREQYEKAYLKSLDHQAVNFKQNDTKALRSKSLLN
SIN3B_MMUS    477
MAPEDQEKLRLDDCLGGTSEVIQRRAIHRIYG-DKAPEVIESLKRNPATAVPVVLKRLKAKEEEWREAQQGFNKIWREQYEKAYLKSLDHQAVNFKQNDTKALRSKSLLN
SIN3B_DRER    530
LSLEDQERFRLDDCLGGTSEVIQRRAVYRIYG-DKAPEIIEGLKRSPATAVPVVLKRLKAKEEEWREAQQGFNKIWREQYEKAYLKSLDHQGVNFKQNDMKALRSKSLLN

SIN3_CELE    1002
QIEQICEERRKNNSTDT-----------------SPHLILEYTPERKVYRDVNDVTGHFF-HDLSGTKCDRDRTKIVL--------FSY--RILMEWLCQEGQQVQIDLD
SIN3_SCER     951
EISSIKVDQTNKKIHWLT-------------PKPKSQLDFDF-PDKNIFYDILCLADTFITHTTAYSNPDKERLKDLLKYFI-SLFFSISFEKIEESLYSHKQNVS-ESS
SIN3A_DMEL  1232
EIETLYDERHDQ--EDD-----------AM-EPFGPHLVLPY-KDKTILDDAANLLIHHVKRQTGIQKQEKQKIKQIIRQFVPDLFFA------------PRQPLS-DDE
SIN3A_HSAP    752
EIESIYDERQEQATEEN-----------AG-VPVGPHLSLAY-EDKQILEDAAALIIHHVKRQTGIQKEDKYKIKQIMHHFIPDLLFA------------QRGDLS-DVE
SIN3A_MMUS    753
EIESIYDERQEQATEEN-----------AG-VPVGPHLSLAY-EDKQILEDAAALIIHHVKRQTGIQKEDKYKIKQIMHHFIPDLLFA------------QRGDLS-DVE
SIN3AA_DRER   755
EIESIFDERQEQVSEDN-----------SSTTASSPHLTLTY-EDHQILEDAAALIIHHVKRQSSINKEDKYKIKQIVYHFIPDMLFA------------QRGVLS-DVE
SIN3AB_DRER   748
EIETLYDERQEQASEDN-----------AA-VPSGPHMTLMY-EDSQILEDAAALIIHHVKRQSGIHKDDKYKIKQIIYHFIPDMLFA------------RRGELS-DVE
SIN3B_HSAP    627
EIESVYDEHQEQHSEGR-----------SA-PSSEPHLIFVY-EDRQILEDAAALISYYVKRQPAIQKEDQGTIHQLLHQFVPSLFFS------------QQLDLG-ASE
SIN3B_MMUS    586
EIESVYDEHQEQHSEGR-----------SA-PSSEPHLIFVY-EDRQILEDAAALISYYVKRQPAIQKEDQGTIRQLLHRFLPSLFFS------------QQCP-G-TSD
SIN3B_DRER    639
EIESIYDERQEQSTEEGSVGQQGRDGTSTA-STSEPHMIFNY-EDKQILEDAASLIIYHVKRQPTIHKDDKDHIKRIIQHFAPDLFFA------------RRGELS-ETE

SIN3_CELE    1084
NGEIFKFQGDLNEDENLMTLL-------------------------------------NMDGRRICGDRVVPVSTSLESNESSI--------------------------
SIN3_SCER    1045
GSD----------DGS------------------------------------------------SIASRKRPYQQEMS-----LLDILHRSRYQKLKRSNDEDGKVPQLS
SIN3A_DMEL  1314
RDDAFPFLVDDNTKMDVDSPLGRTESSTRNAKSTPSESASPARSNASTSSVTPAGIKKETDDSKATTGSFAPASSATASSATPVDDATPSTSSAAAAASAASSSTVSGTE
SIN3A_HSAP    836
EEE----------EEE-------------------MDVDEATGAVKKHNGVGG----------------------------------SPPKSKLLFS-------------
SIN3A_MMUS    837
EEE----------EEE-------------------MDVDEATGAPKKHNGVGG----------------------------------SPPKSKLLFS-------------
SIN3AA_DRER   840
EED----------EEE------------------DMELDE--GASKKHNGLAGGA------------------------------SASPTKSKLLFS-------------
SIN3AB_DRER   832
EED----------EEE-------------------TEPDE--DGTKKHNGT------------------------------------TSSKSKLLFSHT-----------
SIN3B_HSAP    711
ESA----------DED-------------------RDSPQG------------QTTDPSERKKPAPG----P-------------HSSPPEEKGAFGDA-------PATE
SIN3B_MMUS    669
```

```
DSA----------DER----------------DRDRDSAEP------------ERRRPTDEKPPADA-------------------------------------------
SIN3B_DRER   734
EFT----------DEE-------------------GEGEDGAAAGGGVSSTGQQQLNGESRRRRCTS----PQSMDTSTVA----HSINPEGEAVDLR------------


SIN3_CELE   1131
--------------------------------------------------------DHFSENLHQ-KRTRRTFYGDDSVYMIIRYHHMIQERFAKILSTQAIYAQEHFDN
SIN3_SCER   1092
EPPEEEPNTIEEEE----------LIDEEAKNPWLTGNLVE---------------EANSQGIIQNRSIFNL-FANTNIYIFFRHWTTIYERLLEIKQMNERVTKE----
SIN3A_DMEL  1424
GKPKDDPLSSHKEEGAGCTSSGVATSPRQAQDTAGAGVDVEIKLEHPADFSNPKLLPPHAHGQRE-DESYTLFFANNNWYLFLRLHAILCDRLHVMYERARLLAIEEERC
SIN3A_HSAP   870
--------------------------------------------------------NTAAQKLRGMDEVYNLFYVNNNWYIFMRLHQILCLRLLRICSQAERQIEEENRE
SIN3A_MMUS   871
--------------------------------------------------------NTAAQKLRGMDEVYNLFYVNNNWYIFMRLHQILCLRLLRICSQAERQIEEENRE
SIN3AA_DRER  877
--------------------------------------------------------ASTAQRLRSCEDAYNLFYVNNNWYIFLRLHQILCSRLLRLYAQAERQIEEDMRE
SIN3AB_DRER  864
--------------------------------------------------------AASQQKLSSCDDAYNLFFVNNNWYIFLRLHYILCSRLLRIYGQAEKQIEEDTRE
SIN3B_HSAP   756
QPP-----------------------------------------------------LPPPAPHKPLDDVYSLFFANNNWYFFLRLHQTLCSRLLKIYRQAQKQLLEYRTE
SIN3B_MMUS   698
----------------------------------------------------------SPEPPKVLDDVYSLFFANNNWYFFLRLHQTLCARLLKIYRQAQKQLLEHRRE
SIN3B_DRER   795
--------------------------------------------------------DPEAEHQKEMDGVYNLFFVNNNWYFFLRLHQTLCSRLLKIYRQAERQLLEHRAE


SIN3_CELE   1184
QKKNKRWEDGIGADMHGRKALQENIKQRRAAVNDIRNVRSCPSSSYETTLRELKQLGNAQMDIVAFEEAVKNLFPGDIVLFNNIDKLFSSLAKNIHHATCAEERENPIKL
SIN3_SCER   1172
-INTRSTV-TFAKD------LDLLSSQLSEMGLDFVG-----EDAYKQVLRLSRRLINGDLEHQWFEESLRQAYNNKAFKLYTIDKVTQSLVKHAHTLMTDAKTAEIMAL
SIN3A_DMEL  1533
RVNRR-----------ESTATALRLKPKPEIQV---------EDYYPTFLDMLKNVLDGNMDSNTFEDTMREMFGIYAYISFTLDKVVSNAVRQLQYCVTERAALDCVEL
SIN3A_HSAP   924
REWEREVL-GIKRDKSDSPAIQLRLKEPMDVDV---------EDYYPAFLDMVRSLLDGNIDSSQYEDSLREMFTIHAYIAFTMDKLIQSIVRQLQHIVSDEICVQVTDL
SIN3A_MMUS   925
REWEREVL-GIKRDKSDSPAIQLRLKEPMDVDV---------EDYYPAFLDMVRSLLDGNIDSSQYEDSLREMFTIHAYIAFTMDKLIQSIVRQLQHIVSDEVCVQVTDL
SIN3AA_DRER  931
REWEREVL-GLKRDKNENTAVQLRLKEPMDVEV---------EDYYSAFLEMVRNLLDGNMEASQYEDSLREMFTIHAYIAFTMDKLIQNIARQLQHIVSEEICVQVTEL
SIN3AB_DRER  918
REWERNVL-GLKREKNDSPAIQLRLKEPMDIEV---------EDYYSAFLEMVRNLLDGNMETSQYEDQLREMFTIHAYIAFTMDKLIQSIVRQLQHIVSDDICVQVTDL
SIN3B_HSAP   813
KEREKLLC-EGRREKGSDPAMELRLKQPSEVEL---------EEYYPAFLDMVRSLLEGSIDPTQYEDTLREMFTIHAYVGFTMDKLVQNIARQLHHLVSDDVCLKVVEL
SIN3B_MMUS   750
QEREQLLC-EGRREKAADPAMELRLKQPSEVEL---------EEYYPAFLDMVRSLLEGSIDPTQYEDTLREMFTIHAYIGFTMDKLVQNIARQLHHLVSDDVCLKVVEL
SIN3B_DRER   849
QNRERLLMGEGRREKANDLAMELRLKQPSEVEL---------EEYYPAFLDMVRSLLDGNLESTQYEDTLREMFTIHAYIGFTIDKLIQNIVRQLQHLVSDEVCLQVTEL


SIN3_CELE   1294
YLKYRQRIMNA---ERDEDMESVIQEYGQTAEEVLRG-KNTYRFEFVEEQNKPFIKIWVIPREEKDDDDDDDEEGNEGGKDEDNV-------KDEDDGGDGEGRDGPDDD
SIN3_SCER   1269
FVKDRNASTTS---------AKDQIIYRLQVRSHMSNTENMFRIEFDKRTLHV--SIQYIALDDLTLKEPK-ADEDKWKYYVTSY-----ALPHPTEGIPHEKLKIPFLE
SIN3A_DMEL  1623
FATEQRRGCTGGFCRDAHKTFDREMSYQRKAESILNE-ENCFKVYIYKIDCRV--TIELLDSEPEEVDKPAALKAQKFSKYVERL-----ANPALGGGGNTGRSDSALGN
SIN3A_HSAP  1024
YLAENNNGATGGQLNTQNSRSLLESTYQRKAEQLMSD-ENCFKLMFIQSQGQVQLTIELLDTEEENSDDP--VEAERWSDYVERYMNSDTTSPELREHLAQKPVFLPRNL
SIN3A_MMUS  1025
YLAENNNGATGGQLNSQTSRSLLESAYQRKAEQLMSD-ENCFKLMFIQSQGQVQLTVELLDTEEENSDDP--VEAERWSDYVERYMSSDTTSPELREHLAQKPVFLPRNL
SIN3AA_DRER 1031
YLVESGHSSTGGSLLTQSSRAQAEASYQRKAEQLMSD-ENCFKLMFLKSRGNVQLTVELLDTEEENSDGP--MEIERWSDYVMRYLSADFTSPDLKDHLSQKPVFLPRNL
SIN3AB_DRER 1018
YLSECANKATGGSLSTQSSRSNAETSYQRKSEQLMSD-ENCFKLLFQKSKGTVQLAVELLDTEEENSDGP--VDTERWPDYVERYLNTSSASPELREHLSQKPVFLPRNL
SIN3B_HSAP   913
YLNEKKRGAAGGNLSSRCVRAARETSYQWKAERCMAD-ENCFKVMFLQRKGQVIMTIELLDTEEAQTEDP--VEVQHLARYVEQYVGTEGASSSPTEGFLLKPVFLQRNL
SIN3B_MMUS   850
YLNEQQRGAAGGNLSSRCVRAARETSYQWKAERCMAD-ENCFKVMFLQRRGQVIMTIELLDTEEAQTEDP--VEVQHLARYVEQYVGSEGASSSSTEGFLLKPVFLQRNL
SIN3B_DRER   950
YLAERKRGAAGGNLSSQCVRAAWEASYQWKAERVMAE-ENCFKVMFIQNKGQVTLTIELLDTEEAQGDDP--LDVQSLSNYMEQYIGTETLCSQ-AEGYYFKPVFLPRNV


SIN3_CELE   1393
QPP----PSNDDGDDE--EDEDDEED-GPSGADEP---ESTSGSGNVPMDHLNIGENFLWSPPEEKVCTGKM-TTNEKEQRNSVDYMK------VTTTPRLRIHKRM---
SIN3_SCER   1362
RLI----EFGQDIDGT-EVDEEF----SPEGISVS-TLKIKIQPITYQLHIENGSYDVFT----RKATNKYPTIANDNTQKGMVSQKKELISKFLDCAVGLRNNLDEAQK
SIN3A_DMEL  1725
DSV----VDGSDIKTE--ADED------------------TAEHTQSQQWTLLESENEKYI-------------------------------------------------
SIN3A_HSAP  1131
RRI-RKCQRGREQQEK--EGKEGNSKKTMENVDSLDKLECRFKLNSYKMVYVIKSEDYMY----RRTALLR---AHQSHER------------------VSKRLHQRFQA-
SIN3A_MMUS  1132
RRI-RKCQRGREQQEK--EGKEGNSKKTMENVESLDKLECRFKLNSYKMVYVIKSEDYMY----RRTALLR---AHQSHER------------------VSKRLHQRFQA-
SIN3AA_DRER 1138
RRI-RKCQRGCEAQEK--ELKDGERK---ENADSM-KMECMFKLNSYKMVYVFKSEDYMY----RRTALLR---AHQSHER------------------VSTRLHQRFHA-
SIN3AB_DRER 1125
RRI-RKCQRGQEMQMR--EAKETAKK-SSEGGDDS-KMECMFKLNSYKMVYVFKSEDYMY----RRTAQLR---AHQSHQR------------------VSTRLHQRFQM-
SIN3B_HSAP  1020
KKFRRRWQSEQARALRGEARSSWKRL---VGVESACDVDCRFKLSTHKMVFIVNSEDYMY----RRGTLCR---AKQVQPL------------------VLLRHHQHFEE-
SIN3B_MMUS   957
KKF-RRWQCEQVRAMRGEAKSSWKRL---MGVESACDVDCRFRLGTHKMVFIVNSEDYMY----RRGTLCR---AKQVQPL------------------VLLRHHRHFEE-
SIN3B_DRER  1056
RHF-RGWQIKQVEAMR--CRREWHRK---MGVETAGNLDCRFKLNTHKMVFVMNSEDYMY----RRGALVK---ARRSQHR------------------VARAQHERFEQ-

SIN3_CELE   1483 ----------LKEH-----KG-------CNVELMTGFQQLS-------AIVPLM---------------------------
```

```
SIN3_SCER   1458 LSMQKKWEN-LKDSIAKTSAGNQGIESETEKGKITKQEQSDNLDSSTASVLPASITTVPQDDNIETTGNTESS--DKGAKIQ
SIN3A_DMEL  1762 ------WSSPLKIHVQ--------------------------------------------------------SY--------
SIN3A_HSAP  1213 --WVDKW---TKEHVPREMAA-------ETSKWLMGEGLEG--------LVPCTTTCDTETLHFVSINKYRVKY-GTVFKAP
SIN3A_MMUS  1214 --WVDKW---TKEHVPREMAA-------ETSKWLMGEGLEG--------LVPCTTTCDTETLHFVSINKYRVKY-GTVFKAP
SIN3AA_DRER 1216 --WSQRW---AKEHVTRDMAA-------ETSRWLMGEGREG--------LLPCSTSCDPEILHFQRINKYRVKY-GPTNKPQ
SIN3AB_DRER 1205 --WLDRW---RKEHVTSDMAA-------TNHKWLMGEGQDG--------LLPCRTVCEPKILHFQNINKYKVTY-STTS---
SIN3B_HSAP  1102 --WHSRW---LEDNVTVEAAS-------LVQDWLMGEEDED--------MVPCKTLCETVHVHGLPVTRYRVQY-SRRPASP
SIN3B_MMUS  1038 --WHGRW---LEDNVTVAAAG-------LVQDWLMGEEEED--------MVPCKTLCETAHVHGLPVTRYRVQY-SRRPASP
SIN3B_DRER  1135 --WHRGW---LSEHVSPAAER-------SVQDWLMGEEDED--------MIPCKTNCVSLQVKGLHVNRYQVHYNSKAPASP
```

(B) Multiple Sequence Alignment of PAH domain (SIN3 protein)

```
SIN3_CELE_PAH      1 PVYHRFLEIMKDFRAQRIET-------------PDVIEQVAELLYDSPELVLGFNTFL
SIN3_SCER_PAH1     1 DIYNLFLDIMKDFKSQAIDT-------------PGVIERVSTLFRGYPILIQGFNTFL
SIN3A_DMEL_PAH     1 QIYNNFLDIMKEFKSHCIDT-------------PGVIERVSTLFKGHTELIYGFNMFL
SIN3A_HSAP_PAH     1 QVYNDFLDIMKEFKSQSIDT-------------PGVISRVSQLFKGHPDLIMGFNTFL
SIN3A_MMUS_PAH     1 QVYNDFLDIMKEFKSQSIDT-------------PGVISRVSQLFKGHPDLIMGFNTFL
SIN3AA_DRER_PA     1 QVYNDFLDIMKEFKSQSIDT-------------PGVISRVSQLFKGHPDLIMGFNTFL
SIN3AB_DRER_PA     1 QVYNDFLDIMKEFKSQSIDT-------------PGVISRVSQLFKGHPDLIMGFNTFL
SIN3B_DRER_PAH     1 -IYNKFLDIMKEFKSQSIDT-------------PGVINRVSQLFHGHPDLVLGFNAFL
SIN3B_HSAP_PAH     1 ATYNGFLEIMKEFKSQSIDT-------------PGVIRRVSQLFHEHPDLIVGFNAFL
SIN3B_MMUS_PAH     1 ATYNGFLEIMKEFKSQSIDT-------------PGVIRRVSQLFHEHPDLIVGFNAFL
SIN3_SCER_PAH2     1 DIYKHFLEILQTYQREQKPI-------------NEVYAQVTHLFQNAPDLLEDFKKFL
SIN3A_DMEL_PAH     1 AKYKKFLEILHDYQREQKVMKEGSLNQGKMLTEQEVYTQVAKLFGQDEDLLREFGQFL
SIN3A_HSAP_PAH     1 DIYKAFLEILHTYQKEQRNAKEAGGNYTPALTEQEVYAQVARLFKNQEDLLSEFGQFL
SIN3A_MMUS_PAH     1 DIYKAFLEILHTYQKEQRNAKEAGGNYTPALTEQEVYAQVARLFKNQEDLLSEFGQFL
SIN3AB_DRER_PA     1 EVYKSFLEILHTYQKEQRNAKEAGGNYTPTLTEQQVYAQVAQLFKNQEDLLSEFGQFL
SIN3AA_DRER_PA     1 -IYKSFLEILHKYQKEQRNAKEAGGSYTPVLTEQEVYAEVAQLFKNQEDLLSEFGQFL
SIN3B_HSAP_PAH     1 EIYRSFLEILHTYQKEQLNTR---GRPFRGMSEEEVFTEVANLFRGQEDLLSEFGQFL
SIN3B_MMUS_PAH     1 EIYRSFLEILHTYQKEQLHTK---GRPFRGMSEEEVFTEVANLFRGQEDLLSEFGQFL
SIN3B_DRER_PAH     1 ETYRAFLEILHTYQKEQLEVKESRGRSTGGMTEDEVFSKVASLFKGQEDLLAEFGQFL
SIN3_SCER_PAH3     1 --YTEFLKILNLYSQDILDL-------------DDLVEKVDFYLGSNKELFTWFKNFV
SIN3A_DMEL_PAH     1 EVYDNFLRCLTLFNQEIVSK-------------TELLGLVSPFLMKFPDLLRWFTDFL
SIN3A_HSAP_PAH     1 EAYENFLRCLVIFNQEVISR-------------AELVQLVSPFLGKFPELFNWFKNFL
SIN3A_MMUS_PAH     1 EAYENFLRCLVIFNQEVISR-------------AELVQLVSPFLGKFPELFNWFKNFL
SIN3AA_DRER_PA     1 EAYDNFLRCLVIFNEEIISR-------------AELVQLVVPFLGKFPELFTWFKNFL
SIN3AB_DRER_PA     1 EAYDNFLRCLVIFNQEVISR-------------AELVQLVLPFLGKFPELFTWFKNFL
SIN3B_HSAP_PAH     1 EVYENFLRCIALFNQELVSG-------------SELLQLVSPFLGKFPELFAQFKSFL
SIN3B_MMUS_PAH     1 EVYENFLRCIALFNQELVSG-------------SELLQLVSPFLGKFPELFAQFKSFL
SIN3B_DRER_PAH     1 EVYENFLRCIALFNQEVVSG-------------AELLQLVTPFLGKFPELYTQFKSFL
```

(C) Multiple Sequence Alignment of HID domain (SIN3 protein)

```
SIN3_CELE_HID      1 ----LGPSYRFM-KDTKATDCSGRVELDDDLKG--------------------------------VLNDTWTSIPSWSSEDTGSQAIKKSNLEEFHFKTE
SIN3_SCER_HID      1 -CEAFGPSYKRLPKSDTFMPCSGRDDM---CWE--------------------------------VLNDEWVGHPVWASEDSGFIAHRKNQYEETLFKIE
SIN3A_DMEL_HID     1 SCKRLGASYCALPQSTVPKKCSGRTAL---CRE--------------------------------VLNDKWVSFPTWASEDSTFVTSRKTQFEETIYRTE
SIN3A_HSAP_HID     1 SCKRLGSSYRALPKSYQQPKCTGRTPL---CKE--------------------------------VLNDTWVSFPSW-SEDSTFVSSKKTQYEEHIYRCE
SIN3A_MMUS_HID     1 SCKRLGSSYRALPKSYQQPKCTGRTPL---CKE--------------------------------VLNDTWVSFPSW-SEDSTFVSSKKTQYEEHIYRCE
SIN3Ab_DRER_HI     1 SCKRLGSSYRALPKSFQQPRCTGRTPL---CKE--------------------------------VLNDTWVSFPSW-SEDSTFVSSKKTQYEEHIYRCE
SIN3Aa_DRER_HI     1 SCKRLGSSYRALPKSYQLPKCTGRTAI---CKE--------------------------------VLNDTWVSFPSW-SEDSTFVSSKKTQYEEHMYRCE
SIN3B_HSAP_HID     1 SCKRIGSSYRALPKTYQQPKCSGRTAI---CKELDHWTLLQGSWTDDYCMSKFKNTCWIPGYSAGVLNDTWVSFPSW-SEDSTFVSSKKTPYEEQLHRCE
SIN3B_MMUS_HID     1 SCKRIGSSYRALPKTYQQPKCSGRTAI---CKE--------------------------------VLNDTWVSFPSW-SEDSTFVSSKKTPYEEQLHRCE
SIN3B_DRER_HID     1 SCKRLGSSYRALPKTYQQPKCSGRTAI---CKE--------------------------------VLNDTWVSFPSW-SEDSTFVSSKKTPYEEQLHRCE

SIN3_CELE_HID     64 DERYELDIIVDSNRTVIEQLSKTLRDYEAMSDEDKKS
SIN3_SCER_HID     65 EERHEYDFYIESNLRTIQCLETIVNKIENMTENEKAN
SIN3A_DMEL_HID    66 DERFELDLVIEVNSATIRVLENLQKKMSRMSTEELSK
SIN3A_HSAP_HID    65 DERFELDVVLETNLATIRVLEAIQKKLSRLSAEEQAK
SIN3A_MMUS_HID    65 DERFELDVVLETNLATIRVLEAIQKKLSRLSAEEQAK
SIN3Ab_DRER_HI    65 DERFELDVVLETNLATIRVLEAVQKRISRMSAEEQAK
SIN3Aa_DRER_HI    65 DERFELDVVLETNLATIRVLETTQKKLSRMSAEEQAK
SIN3B_HSAP_HID    97 DERFELDVVLETNLATIRVLESVQKKLSRMAPEDQEK
SIN3B_MMUS_HID    65 DERFELDVVLETNLATIRVLESVQKKLSRMAPEDQEK
SIN3B_DRER_HID    65 DERFELDVVLETNLATIRVLESVQKKLSRLSLEDQER
```

(D) Multiple Sequence Alignment of Sin3A C domain (SIN3 protein)

```
SIN3_CELE_Sin3    1 FYGDDSVYMIIRYHHMIQERFAKILSTQAIYAQEHFDNQKKNKRWEDGIGADMHGRKALQENIKQRRAAVNDIRNVRSCPSSSYETTLRELKQLGNAQMD
SIN3_SCER_Sin3    1 LFANTNIYIFFRHWTTIYERLLEIKQMNERVTKE-----INTRSTV-TFAKD------LDLLSSQLSEMGLDFVG-----EDAYKQVLRLSRRLINGDLE
SIN3A_DMEL_Sin    1 FFANNNWYLFLRLHAILCDRLHVMYERARLLAIEEERCRVNRR-----------ESTATALRLKPKPEIQV---------EDYYPTFLDMLKNVLDGNMD
SIN3A_HSAP_Sin    1 FYVNNNWYIFMRLHQILCLRLLRICSQAERQIEEENREREWEREVL-GIKRDKSDSPAIQLRLKEPMDVDV---------EDYYPAFLDMVRSLLDGNID
SIN3A_MMUS_Sin    1 FYVNNNWYIFMRLHQILCLRLLRICSQAERQIEEENREREWEREVL-GIKRDKSDSPAIQLRLKEPMDVDV---------EDYYPAFLDMVRSLLDGNID
SIN3AA_DRER_Si    1 FYVNNNWYIFLRLHQILCSRLLRLYAQAERQIEEDMREREWEREVL-GLKRDKNENTAVQLRLKEPMDVEV---------EDYYSAFLEMVRNLLDGNME
SIN3AB_DRER_Si    1 FFVNNNWYIFLRLHYILCSRLLRIYGQAEKQIEEDTREREWERNVL-GLKREKNDSPAIQLRLKEPMDIEV---------EDYYSAFLEMVRNLLDGNME
SIN3B_HSAP_Sin    1 FFANNNWYFFLRLHQTLCSRLLKIYRQAQKQLLEYRTEKEREKLLC-EGRREKGSDPAMELRLKQPSEVEL---------EEYYPAFLDMVRSLLEGSID
SIN3B_MMUS_Sin    1 FFANNNWYFFLRLHQTLCARLLKIYRQAQKQLLEHRREQEREQLLC-EGRREKAADPAMELRLKQPSEVEL---------EEYYPAFLDMVRSLLEGSID
SIN3B_DRER_Sin    1 FFVNNNWYFFLRLHQTLCSRLLKIYRQAERQLLEHRAEQNRERLLMGEGRREKANDLAMELRLKQPSEVEL---------EEYYPAFLDMVRSLLDGNLE

SIN3_CELE_Sin3  101 IVAFEEAVKNLFPGDIVLFNNIDKLFSSLAKNIHHATCAEERENPIKLYLKYRQRIMNA---ERDEDMESVIQEYGQTAEEVLRG-KNTYRFEFVEEQNK
SIN3_SCER_Sin3   84 HQWFEESLRQAYNNKAFKLYTIDKVTQSLVKHAHTLMTDAKTAEIMALFVKDRNASTTS---------AKDQIIYRLQVRSHMSNTENMFRIEFDKRTLH
SIN3A_DMEL_Sin   81 SNTFEDTMREMFGIYAYISFTLDKVVSNAVRQLQYCVTERAALDCVELFATEQRRGCTGGFCRDAHKTFDREMSYQRKAESILNE-ENCFKVYIYKIDCR
SIN3A_HSAP_Sin   91 SSQYEDSLREMFTIHAYIAFTMDKLIQSIVRQLQHIVSDEICVQVTDLYLAENNNGATGGQLNTQNSRSLLESTYQRKAEQLMSD-ENCFKLMFIQSQGQ
SIN3A_MMUS_Sin   91 SSQYEDSLREMFTIHAYIAFTMDKLIQSIVRQLQHIVSDEVCVQVTDLYLAENNNGATGGQLNSQTSRSLLESAYQRKAEQLMSD-ENCFKLMFIQSQGQ
SIN3AA_DRER_Si   91 ASQYEDSLREMFTIHAYIAFTMDKLIQNIARQLQHIVSEEICVQVTELYLVESGHSSTGGSLLTQSSRAQAEASYQRKAEQLMSD-ENCFKLMFLKSRGN
SIN3AB_DRER_Si   91 TSQYEDQLREMFTIHAYIAFTMDKLIQSIVRQLQHIVSDDICVQVTDLYLSECANKATGGSLSTQSSRSNAETSYQRKSEQLMSD-ENCFKLLFQKSKGT
SIN3B_HSAP_Sin   91 PTQYEDTLREMFTIHAYVGFTMDKLVQNIARQLHHLVSDDVCLKVVELYLNEKKRGAAGGNLSSRCVRAARETSYQWKAERCMAD-ENCFKVMFLQRKGQ
SIN3B_MMUS_Sin   91 PTQYEDTLREMFTIHAYIGFTMDKLVQNIARQLHHLVSDDVCLKVVELYLNEQQRGAAGGNLSSRCVRAARETSYQWKAERCMAD-ENCFKVMFLQRRGQ
SIN3B_DRER_Sin   92 STQYEDTLREMFTIHAYIGFTIDKLIQNIVRQLQHLVSDEVCLQVTELYLAERKRGAAGGNLSSQCVRAAWEASYQWKAERVMAE-ENCFKVMFIQNKGQ

SIN3_CELE_Sin3  197 ----------------------------------------------------------------------------------------------------
SIN3_SCER_Sin3  175 V--SIQYIALDDLTLKEPK-ADEDKWKYYVTSY-----ALPHPTEGIPHEKLKIPFLERLI----EFGQDI-----DGTEVDEEFSPEGISVS-TLKIKI
SIN3A_DMEL_Sin  180 V--TIELLDSEPEEVDKPAALKAQKFSKYVER-------------------------------------------------------------------
SIN3A_HSAP_Sin  190 VQLTIELLDTEEENSDDP--VEAERWSDYVERYMNSDTTSPELREHLAQKPVFLPRNLRRI-RKCQRGREQQEK--EGKEGNSKKTMENVDSLDKLECRF
SIN3A_MMUS_Sin  190 VQLTVELLDTEEENSDDP--VEAERWSDYVERYMSSDTTSPELREHLAQKPVFLPRNLRRI-RKCQRGREQQEK--EGKEGNSKKTMENVESLDKLECRF
SIN3AA_DRER_Si  190 VQLTVELLDTEEENSDGP--MEIERWSDYVMRYLSADFTSPDLKDHLSQKPVFLPRNLRRI-RKCQRGCEAQEK--ELKDGERK---ENADSM-KMECMF
SIN3AB_DRER_Si  190 VQLAVELLDTEEENSDGP--VDTERWPDYVERYLNTSSASPELREHLSQKPVFLPRNLRRI-RKCQRGQEMQMR--EAKETAKK-SSEGGDDS-KMECMF
SIN3B_HSAP_Sin  190 VIMTIELLDTEEAQTEDP--VEVQHLARYVEQYVGTEGASSSPTEGFLLKPVFLQRNLKKFRRRWQSEQARALRGEARSSWKRL---VGVESACDVDCRF
SIN3B_MMUS_Sin  190 VIMTIELLDTEEAQTEDP--VEVQHLARYVEQYVGSEGASSSSTEGFLLKPVFLQRNLKKF-RRWQCEQVRAMRGEAKSSWKRL---MGVESACDVDCRF
SIN3B_DRER_Sin  191 VTLTIELLDTEEAQGDDP--LDVQSLSNYMEQYIGTETLCSQA-EGYYFKPVFLPRNVRHF-RGWQIKQVEAMR--CRREWHRK---MGVETAGNLDCRF

SIN3_CELE_Sin3      -----------------
SIN3_SCER_Sin3  257 QPITYQLHIENGSYDVF
SIN3A_DMEL_Sin      -----------------
SIN3A_HSAP_Sin  285 KLNSYKMVYVIKSEDYM
```

Fig. S3: Orthogroups of *C. elegans* proteins. The spider plots indicate the number of proteins in different species; *C. elegans* specific pathways like DNA damage pathway (A), JNK-MEK pathway (B), ERK-MAPK pathway (C), p38-MAPK pathway (D), insulin/IGF signalling pathway (E), Catalases (F) and Superoxide dismutases (G) are indicated at the top of each spider plot. Hsap, *Homo sapiens*; Mmus, *Mus musculus*; Drer, *Danio rerio*; Dmel, *Drosophila melanogaster*; Scer, *Saccharomyces cerevisiae*; Cele, *C. elegans*.

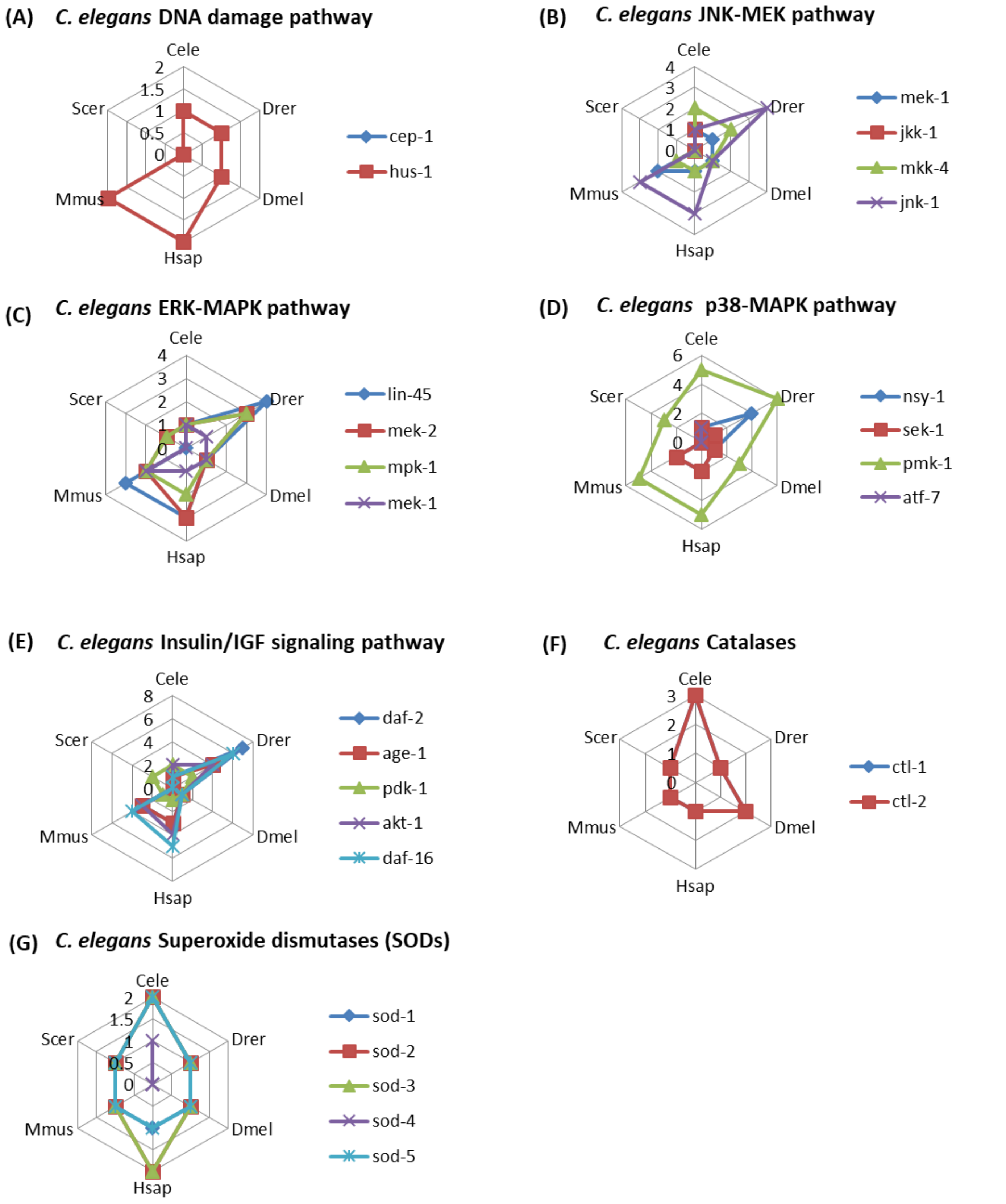

Fig. S4: Protein interactome of SIN3 orthologs gives an overview of the protein-protein interactions derived from the integrated interaction database GeneMANIA (*Caenorhabditis elegans*, *Saccharomyces cerevisiae*, *Drosophila melanogaster*, *Danio rerio*, *Homo sapiens*, *Mus musculus*). The network images for physical interaction were obtained (maximum resultant genes = 100) using Automatically selected Weighting Method (default)- Gene-Ontology (GO) based weighting, Biological Process based (for <5 query genes).

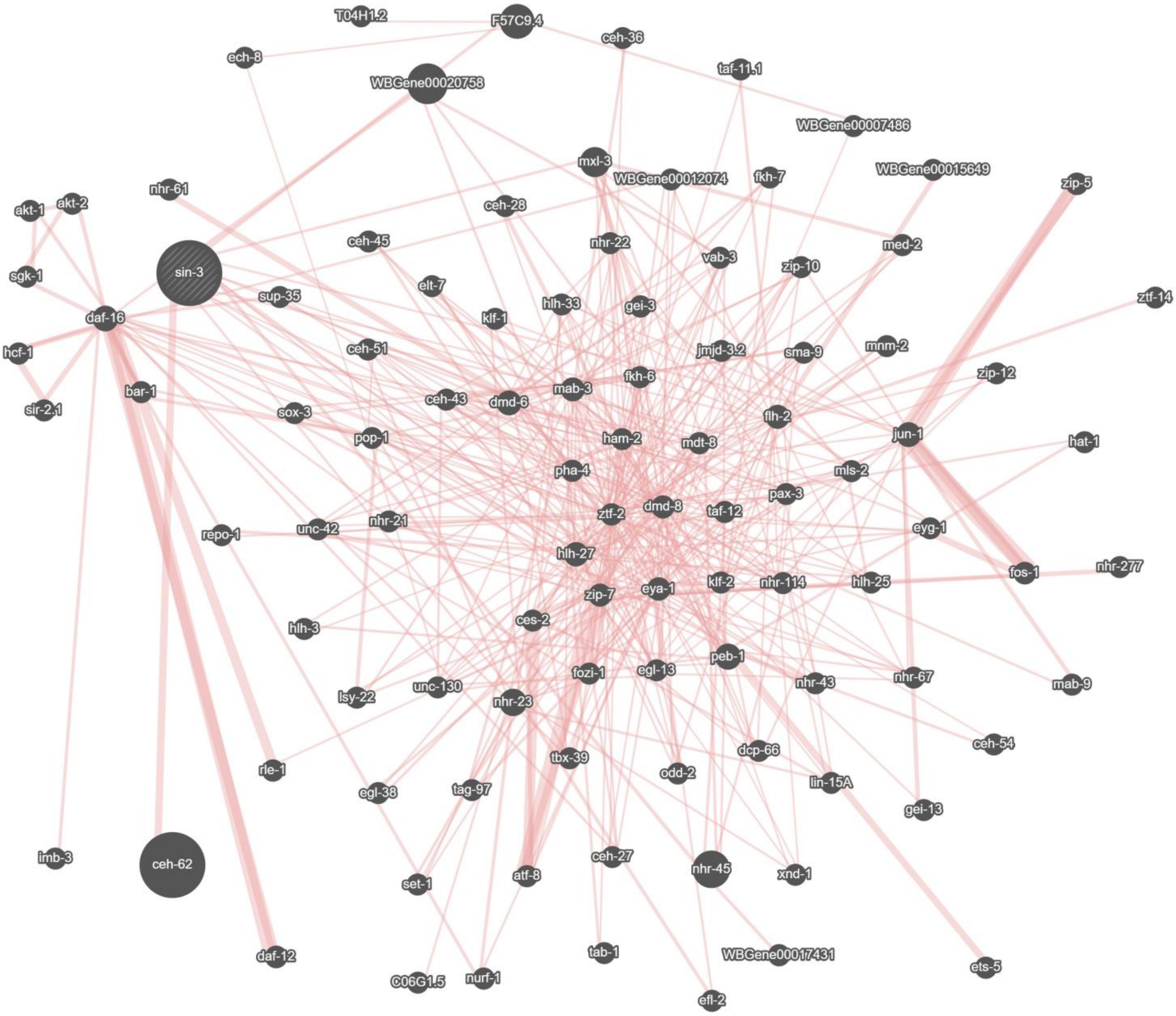

Caenorhabditis elegans

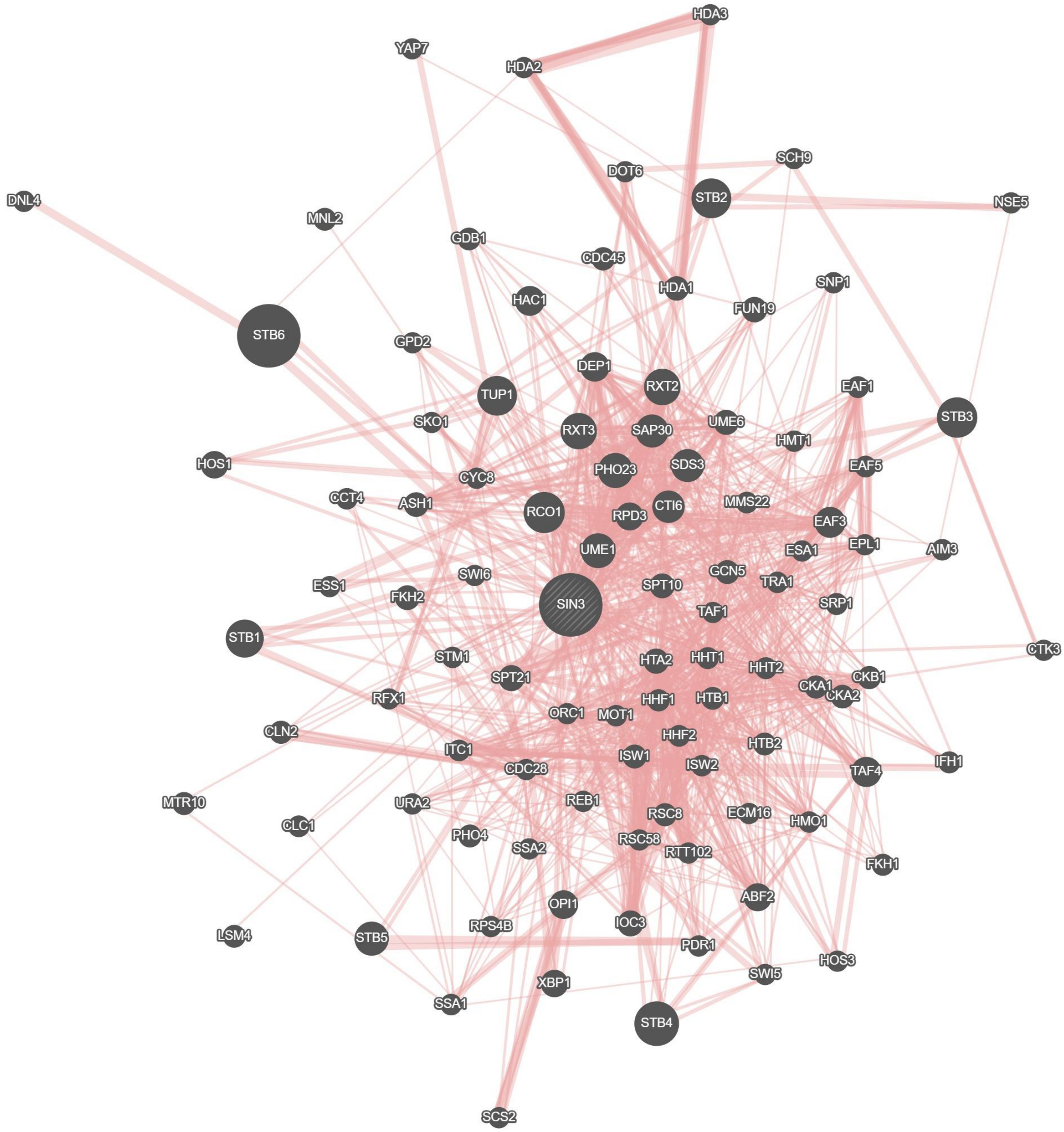

Saccharomyces cerevisiae

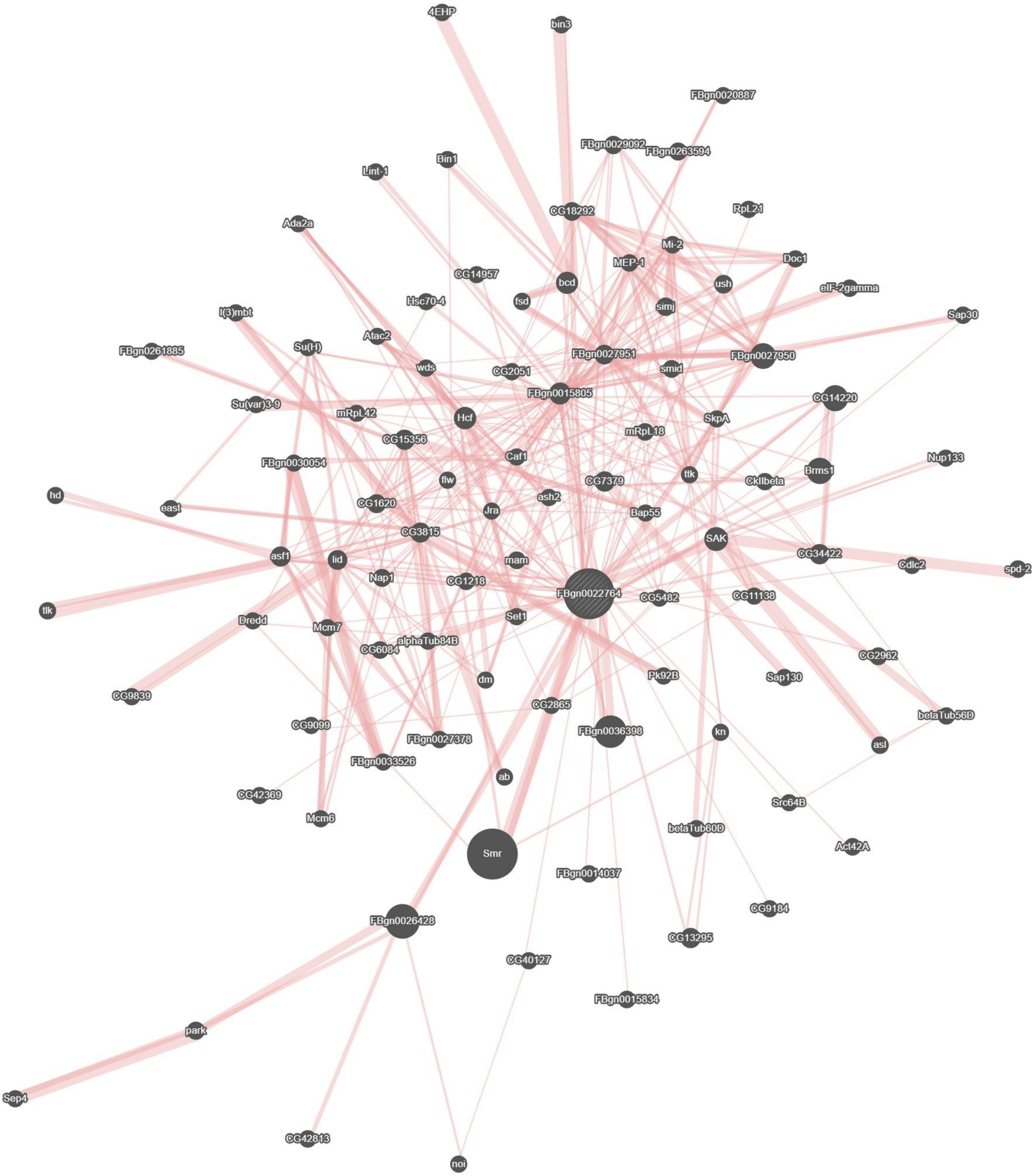

Drosophila melanogaster

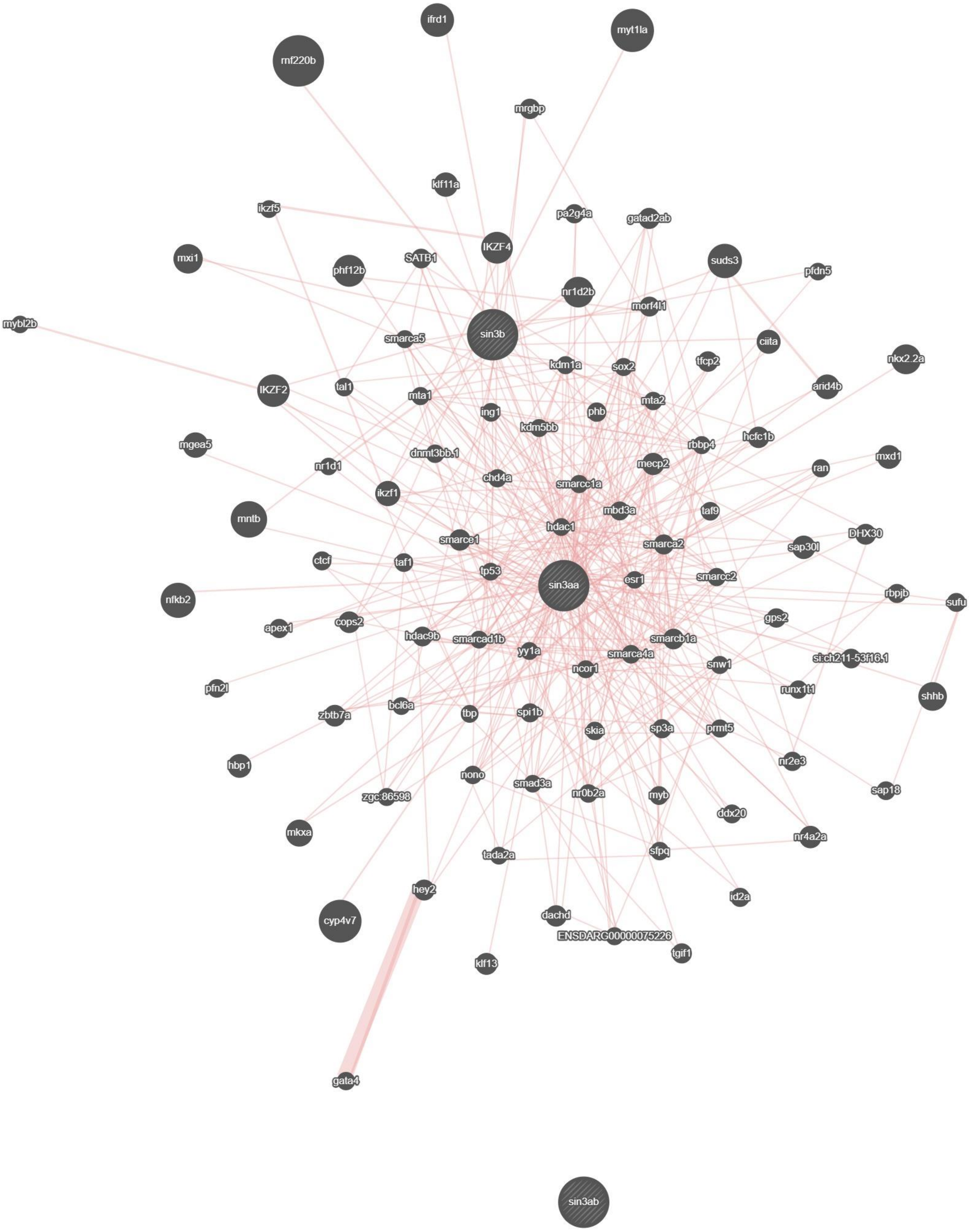
ifrd1
myt1la
rnf220b
mrgbp
klf11a
ikzf5
pa2g4a
gatad2ab
IKZF4
mxi1
SATB1
phf12b
suds3
pfdn5
nr1d2b
morf4l1
mybl2b
smarca5
sin3b
ciita
kdm1a
sox2
tfcp2
nkx2.2a
IKZF2
tal1
mta1
mta2
arid4b
ing1
phb
kdm5bb
hcfc1b
mgea5
rbbp4
dnmt3bb.1
mxd1
nr1d1
mecp2
ran
chd4a
smarcc1a
ikzf1
mbd3a
taf9
mntb
hdac1
DHX30
smarce1
smarca2
sap30l
ctcf
taf1
tp53
sin3aa
esr1
smarcc2
nfkb2
rbpjb
sufu
gps2
apex1
cops2
hdac9b
smarcad1b
smarcb1a
yy1a
smarca4a
si:ch211-53f16.1
ncor1
snw1
pfn2l
runx1t1
shhb
bcl6a
zbtb7a
tbp
spi1b
skia
sp3a
prmt5
hbp1
nr2e3
nono
smad3a
nr0b2a
myb
sap18
zgc:86598
ddx20
mkxa
nr4a2a
sfpq
tada2a
hey2
id2a
cyp4v7
dachd
ENSDARG00000075226
tgif1
klf13
gata4
sin3ab

Danio rerio

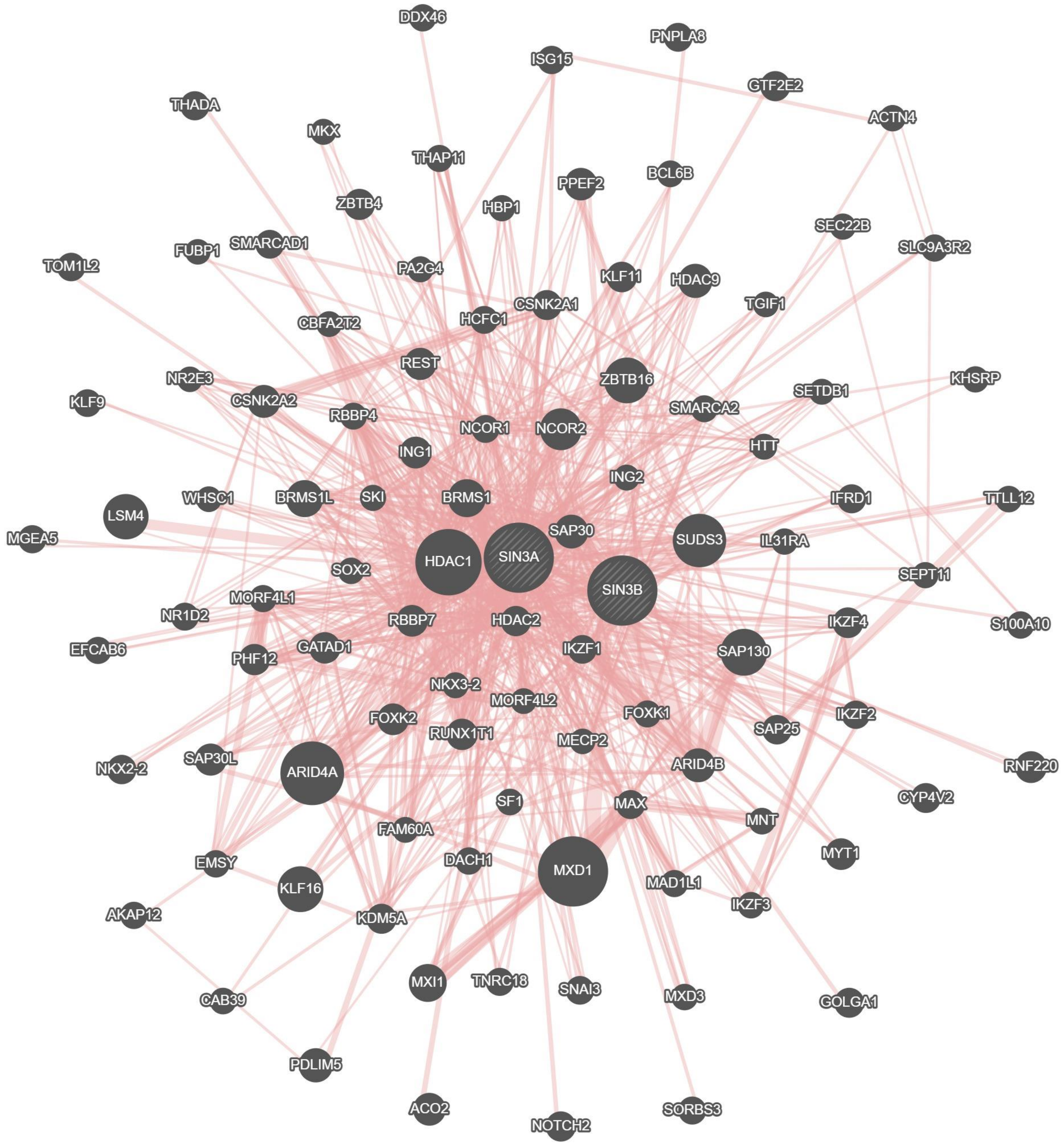

Homo sapiens

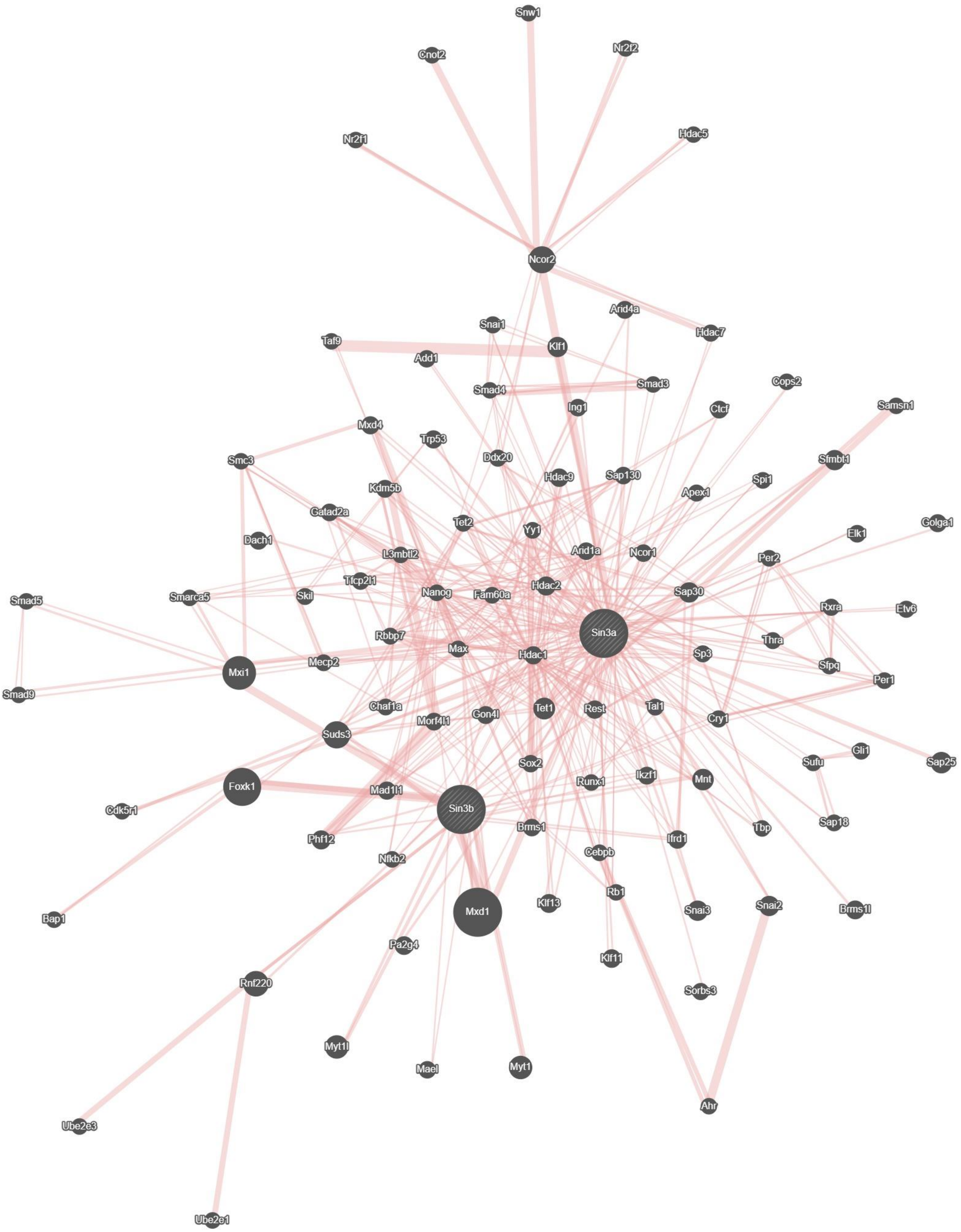

Mus musculus

Fig. S5: Protein interactome of the *C. elegans* SIN-3 protein constructed using Cytoscape. The network image for direct physical interaction was obtained using manually curated direct protein interaction evidence. The squares in the figure segregate the interactome data based on literature evidence for protein-protein interaction.

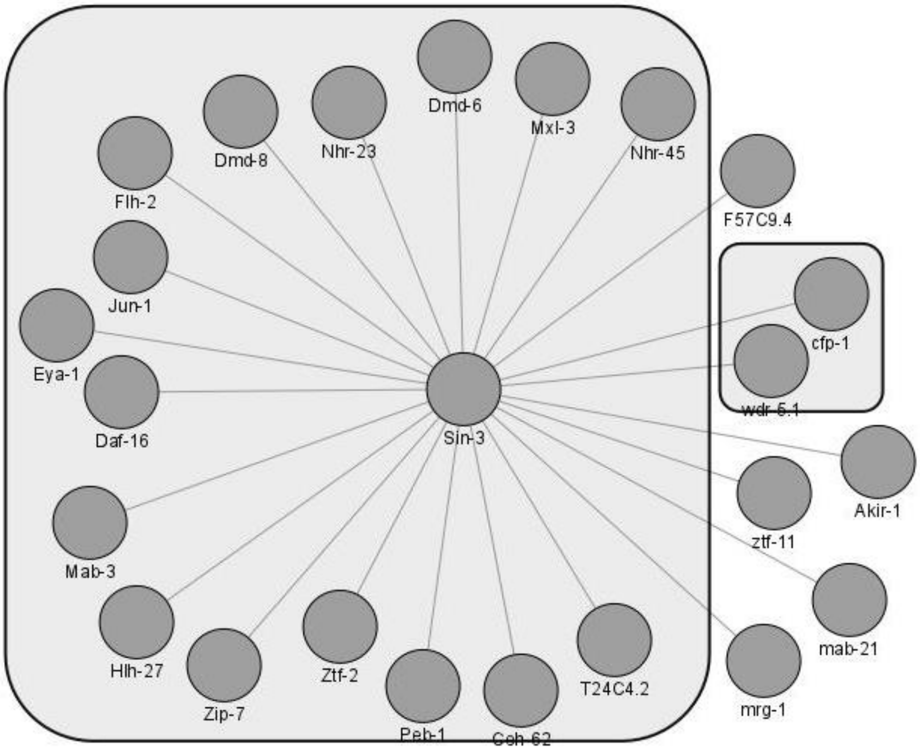

Fig. S6: Heatmap distribution of the abundance of proteins involved in the (A) GeneMANIA interactome, and (B) manually curated interactome of Sin-3 protein in *C. elegans* (Cele) compared to other model organisms (Hsap, *Homo sapiens*; Mmus, *Mus musculus*; Drer, *Danio rerio*; Dmel, *Drosophila melanogaster*; Scer, *Saccharomyces cerevisiae*). Each entry indicates the number of proteins per proteome. Color scale key is depicted at the side.

(A)

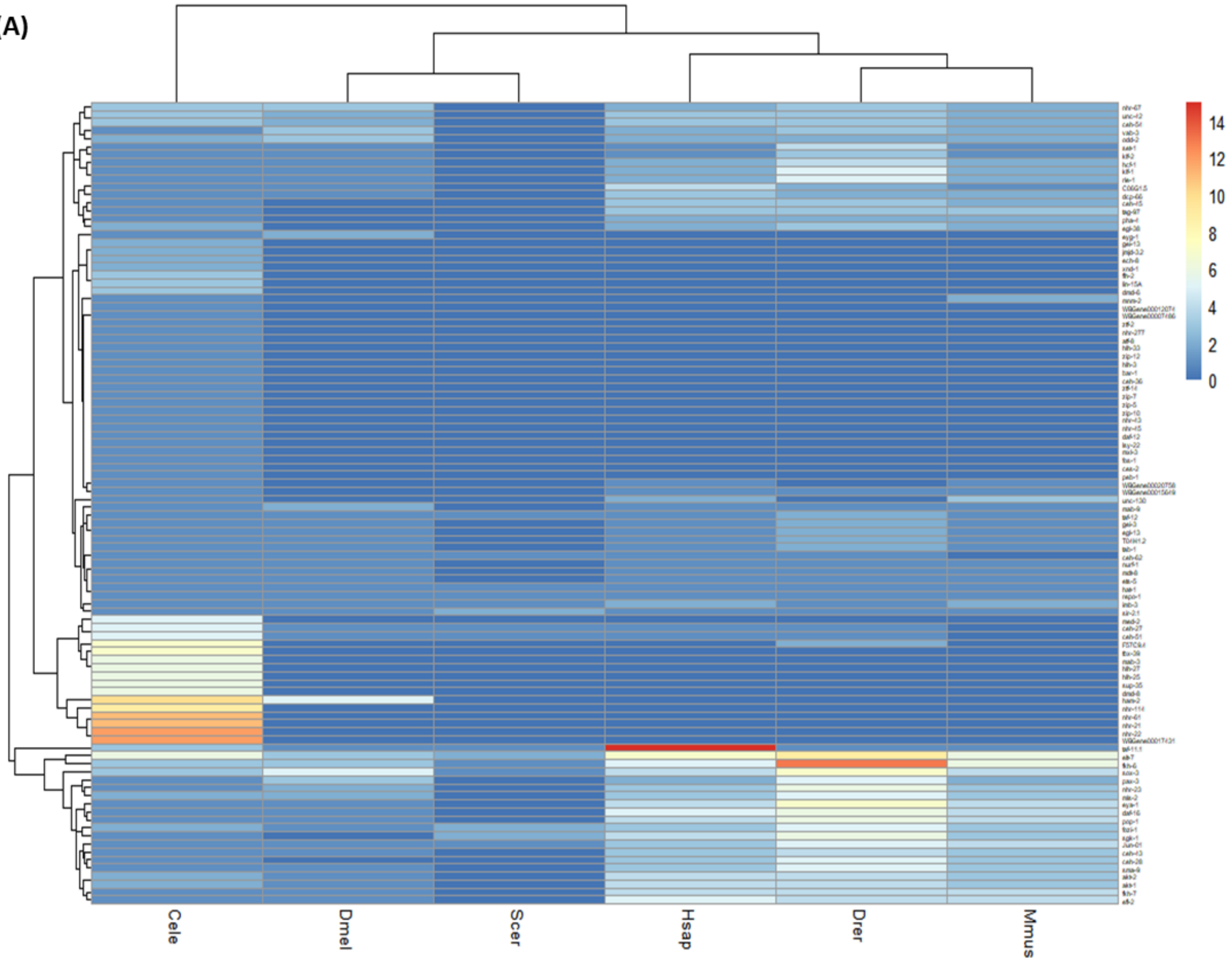

(B)

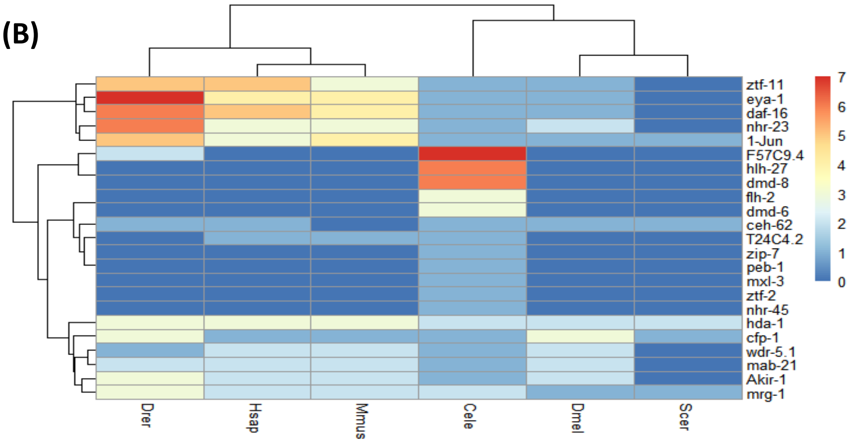

Supplementary Tables

Table S1: Protein-protein interactions of the SIN-3 protein unique to *C. elegans* GeneMANIA network.

**Sin-3 protein interactors unique to *C. elegans***							
ATF-8	DAF-16	EYG-1	HLH-25	MLS-2	NURF-1	SMA-9	WBGENE00015649
BAR-1	DMD-6	F57C9.4	HLH-27	MNM-2	ODD-2	SUP-35	WBGENE00017431
C06G1.5	DMD-8	FKH-6	HLH-33	NHR-114	PAX-3	T04H1.2	WBGENE00020758
CEH-36	ECH-8	FKH-7	IMB-3	NHR-21	PEB-1	TAF-11.1	XND-1
CEH-43	EFL-2	FLH-2	KLF-1	NHR-22	PHA-4	TAF-12	ZIP-10
CEH-45	EGL-13	FOZI-1	LIN-15A	NHR-277	POP-1	TBX-39	ZIP-12
CEH-51	EGL-38	GEI-13	LSY-22	NHR-43	REPO-1	UNC-42	ZIP-5
CEH-54	ELT-7	GEI-3	MAB-3	NHR-45	RLE-1	VAB-3	ZIP-7
CES-2	ETS-5	HAM-2	MAB-9	NHR-61	SET-1	WBGENE00007486	ZTF-2
DAF-12	EYA-1	HAT-1	MDT-8	NHR-67	SIR-2.1	WBGENE00012074	

Table S2: List of unaligned gap sequences in *C. elegans* SIN-3 protein post Multiple Sequence Alignment of Sin-3 orthologs (Gln, Glutamine; Asp, Aspartate).

**Start**	**End**	**Sequence**	**Motifs present**
3	197	PPPGGGGGNNGGDQSQQQPTNNATLFLLQMIQQSQHQQQHQNQQQQQLELQIRDQERI LIEQQRMQHQQQQNQLLQGLNQFPFNPLGLFQVQAAVQAAQAQFAQNAQGSPIPFHIGS PLQPSHSPAASALQQQYLLPSHSPAITPFARNSEAARNIEQFIAQEEAANVPRANSQQQSP LIRPIPQQQALNIQNL	Gln rich
211	230	RQVPVQQVQHQQHIPTPPLA	Gln rich
267	278	GGRRQNRPGRRK	
369	436	QMQPRVLLSPDERRARAIEAGAQAVGAIELGSQEGISKDEDRTIEDEDMDKSKEKDDVD GIDDEDDEE	Asp rich
450	483	EDNHLIEEIICDDRKKDDCEDSQQEIEMSSELAA	Asp rich
523	581	MEKLRKDDEDDEIEENEKIEVDDVPGPSNAPQEIKKPDDIEKKDSSKNLQIEESCSDYL	
582	669	VSMLANCCIGEPDLLAATIDFLPYLGKLLVNGSDAIALKIKTILHFSATNDRNDIPPVNRV NPSDVDMDLVKQMEKCKMGTKKNEKLKLK	
766	790	AVMRKDLPAIQPKRGLRDQKMLQQV	
1062	1139	FSYRILMEWLCQEGQQVQIDLDNGEIFKFQGDLNEDENLMTLLNMDGRRICGDRVVPVS TSLESNESSIDHFSENLHQ	
215	225	VNDIRNVRSCP	
1361	1404	DEEGNEGGKDEDNVKDEDDGGDGEGRDGPDDDQPPPSNDDGDDE	Asp rich
1413	1507	GPSGADEPESTSGSGNVPMDHLNIGENFLWSPPEEKVCTGKMTTNEKEQRNSVDYMKV TTTPRLRIHKRMLKEHKGCNVELMTGFQQLSAIVPLM	

Table S3: List of *C. elegans* SIN-3 protein interactors with Glutamine/Aspartate repeats.

S. No.	Protein Names	Accession No.	prf:GLN RICH	prf:ASP RICH
1	DCP-66	G5ED89.2	280-398, 587-711	
2	FOZI-1	CAA80128.1	89-134	
3	TAF-12	CAB60514.2	35-217	
4	GEI-13	CAA80171.2	290-387	
5	SMA-9	CAF31491.2	13-450	1033-1053
6	CEH-36	Q93352.3	174-185	
